# The genetic landscape of mitochondrial diseases in the next-generation sequencing era: a Portuguese cohort study

**DOI:** 10.3389/fcell.2024.1331351

**Published:** 2024-02-23

**Authors:** C. Nogueira, C. Pereira, L. Silva, Mateus Laranjeira, A. Lopes, R. Neiva, E. Rodrigues, T. Campos, E. Martins, A. Bandeira, M. Coelho, M. Magalhães, J. Damásio, A. Gaspar, P Janeiro, A. Levy Gomes, A. C. Ferreira, S. Jacinto, J. P. Vieira, L. Diogo, H. Santos, C. Mendonça, L. Vilarinho

**Affiliations:** ^1^ Research & Development Unit, Human Genetics Department, National Institute of Health Doutor Ricardo Jorge, Lisbon, Portugal; ^2^ Newborn Screening, Metabolism & Genetics Unit, Human Genetics Department, National Institute of Health Doutor Ricardo Jorge, Lisbon, Portugal; ^3^ Inherited Metabolic Diseases Reference Centre, São João Hospital University Centre, Porto, Portugal; ^4^ Inherited Metabolic Diseases Reference Centre, Santo António Hospital University Centre, Porto, Portugal; ^5^ Neurology Department, Santo António Hospital University Centre, Porto, Portugal; ^6^ Inherited Metabolic Diseases Reference Centre, Lisboa Norte Hospital University Centre, Lisboa, Portugal; ^7^ Neurology Department, Lisboa Norte Hospital University Centre, Lisboa, Portugal; ^8^ Inherited Metabolic Diseases Reference Centre, Lisboa Central Hospital Centre, Lisboa, Portugal; ^9^ Inherited Metabolic Diseases Reference Centre, Coimbra Hospital and University Centre, Coimbra, Portugal; ^10^ Inherited Metabolic Diseases Reference Centre, Vila Nova de Gaia Hospital Centre, Vila Nova de Gaia, Portugal; ^11^ Pediatric Department, Faro Hospital and University Centre, Faro, Portugal

**Keywords:** mitochondrial diseases, next-generation sequencing, mitochondrial DNA, nuclear DNA, nuclear genes, respiratory chain, oxidative phosphorylation

## Abstract

**Introduction:** Rare disorders that are genetically and clinically heterogeneous, such as mitochondrial diseases (MDs), have a challenging diagnosis. Nuclear genes codify most proteins involved in mitochondrial biogenesis, despite all mitochondria having their own DNA. The development of next-generation sequencing (NGS) technologies has revolutionized the understanding of many genes involved in the pathogenesis of MDs. In this new genetic era, using the NGS approach, we aimed to identify the genetic etiology for a suspected MD in a cohort of 450 Portuguese patients.

**Methods:** We examined 450 patients using a combined NGS strategy, starting with the analysis of a targeted mitochondrial panel of 213 nuclear genes, and then proceeding to analyze the whole mitochondrial DNA.

**Results and Discussion:** In this study, we identified disease-related variants in 134 (30%) analyzed patients, 88 with nuclear DNA (nDNA) and 46 with mitochondrial DNA (mtDNA) variants, most of them being pediatric patients (66%), of which 77% were identified in nDNA and 23% in mtDNA. The molecular analysis of this cohort revealed 72 already described pathogenic and 20 novel, probably pathogenic, variants, as well as 62 variants of unknown significance. For this cohort of patients with suspected MDs, the use of a customized gene panel provided a molecular diagnosis in a timely and cost-effective manner. Patients who cannot be diagnosed after this initial approach will be further selected for whole-exome sequencing.

**Conclusion:** As a national laboratory for the study and research of MDs, we demonstrated the power of NGS to achieve a molecular etiology, expanding the mutational spectrum and proposing accurate genetic counseling in this group of heterogeneous diseases without therapeutic options.

## 1 Introduction

Through the oxidative phosphorylation (OXPHOS) process, mitochondria play a crucial role in producing over 90% of the cell’s energy in the form of adenosine triphosphate (ATP), earning them the nickname “powerhouses of the cell.” Distinguished by their unique features, mitochondria possess their own genetic material, referred to as mitochondrial DNA (mtDNA), which is formed by a double-stranded circular molecule of 16,569 base pairs. This genome is formed by 37 genes responsible for encoding 13 polypeptides vital for facilitating protein synthesis within the organelle, 22 tRNAs, and 2 rRNAs. However, the majority of the remaining 1,500 mitochondrial proteins are encoded by nuclear genes, which are initially translated in the cytoplasm before being subsequently imported into the mitochondrion (Friedman and Nunnari, 2014; Mishra and Chari, 2014; Kotrys and Szczesny, 2019).

Mitochondrial diseases (MDs) have become a prevalent cause of inherited metabolic diseases, impacting approximately 1 in 5,000 individuals (Gorman et al., 2015) and revealing a high genetic heterogeneity as they can result from pathogenic variants in either the nuclear DNA (nDNA) or mtDNA. Consequently, a MD can manifest through various inheritance patterns, from an autosomal dominant or recessive pattern to X-linked, *de novo* or maternal inheritance (Rahman and Rahman, 2018). Morbimortality rates among the affected individuals vary significantly due to this heterogeneous nature, which tends to be higher in cases with a rapidly progressive pattern and a pediatric onset (Gorman et al., 2016).

High-energy-demand tissues such as the brain, muscles, peripheral nerves, eyes, and heart tend to be the most affected tissues when an impairment of mitochondrial function is present. Clinically, this tends to result in multisystem involvement, although certain patients can only exhibit involvement in a single system, usually the neurological system (DiMauro et al., 2013; Maresca et al., 2020).

In the pre-molecular era, the diagnosis of a MD traditionally depended on invasive techniques, such as tissue biopsies for histochemical and biochemical analysis. With the description of the first disease-causing mutation in the mtDNA in 1988, the molecular era of mitochondrial medicine began (Holt et al., 1988; Wallace et al., 1988; Zeviani et al., 1988).

In this molecular era, next-generation sequencing (NGS) technology can generate an enormous amount of sequence data in a short time at an affordable cost, making this approach ideal for the analysis of large patient cohorts (Stenton and Prokisch, 2020), allowing the identification of pathogenic mutations affecting both the mitochondrial and nuclear genomes (Riley et al., 2020), and shortening the diagnostic odyssey (Schon et al., 2020). Thus, NGS strategies not only allow the identification of known pathogenic variants but also enable the identification of suspected pathogenic variants in the candidate genes, novel variants in the mitochondrial disease-related genes, several variants of unknown significance (VUS), and even variants in disease genes not yet associated with MD but presenting similar phenotypes (Schlieben and Prokisch, 2020). To date, the causes of mitochondriopathies have been recognized to originate from pathogenic variants in more than 400 genes in both the mitochondrial and nuclear DNA (Frazier et al., 2019; Falk, 2020; Rahman, 2020; Stenton and Prokisch, 2020).

Providing these patients and their families with a definitive genetic diagnosis offers significant advantages, whether by facilitating genetic counseling, enabling personalized information about the treatment and prognosis, or providing access to reproductive options, including prenatal diagnosis (French et al., 2019; Poulton et al., 2019).

The primary aim of this study was to determine the genetic basis of the patients clinically and/or biochemically suspected of MDs in the NGS era and how this diagnostic tool has remarkably reduced the cost and increased the availability to reach a definitive diagnosis in a short time. This work, covering more than 304 patients, complemented the previous study of 146 patients already published (Nogueira et al., 2019) and allowed the evaluation of the phenotypic characteristics and genetic landscape of 450 MD patients in Portugal.

## 2 Patients and methods

### 2.1 Patients

We examined 450 patients, comprising 219 males and 231 females, with 262 in the pediatric group and 188 in the adult group, across seven Portuguese hospitals. These individuals, lacking a molecular diagnosis, were referred to our laboratory when the clinical, biochemical, and/or neuroimaging findings suggested a MD, following the criteria established by the Mitochondrial Medicine Society (Parikh et al., 2015). Notably, 146 out of the 450 patients had previously undergone a comprehensive investigation in our national laboratory. This involved traditional Sanger sequencing of the most prevalent mtDNA mutations and a group of genes linked to specific biochemical defects and/or clinical presentations. Most of the patients underwent metabolic evaluation, which included the analysis of the redox state, amino acids, organic acids, and acylcarnitines. Mitochondrial respiratory chain (MRC), multiple deletions, and/or mtDNA depletion were also investigated in 115/450 (26%) patients suspicious of mtDNA maintenance defects.

Approval for this study was granted by the Ethics Committee of the National Institute of Health Doutor Ricardo Jorge and the Local Ethics Committee of the participating hospitals. In accordance with the Declaration of Helsinki, informed consent for genetic studies was obtained from all examined patients or, when applicable, from their relatives.

### 2.2 Methods

#### 2.2.1 DNA/RNA isolation

Bio Robot EZ1 (QIAGEN, Hilden, Germany) was used to extract genomic DNA from peripheral blood samples following the standard procedures.

In accordance with the manufacturer’s recommendations, total RNA was extracted using the PAXgene Blood RNA Kit (PreAnalytiX, QIAGEN, Germantown, MD, United States), and the quantification of both DNA and RNA was performed using a NanoDrop 2000 C spectrophotometer (Thermo Fisher Scientific, Waltham, MA, United States).

#### 2.2.2 Next-generation sequencing

##### 2.2.2.1 Targeted nuclear panel

For DNA studies, we employed a NGS-guided strategy performed on a MiSeq Illumina platform. Using a SureSelect XT HS Kit (Agilent Technologies, Santa Clara, CA, United States), a set of targeted nuclear gene panels designed with SureDesign software (Agilent Technologies) was captured. This panel covered 213 nuclear genes, 188 of which were known to be associated with MDs, while 25 of these consisted of candidate genes based on their involvement in various pathways or the capability of their clinical presentations to mimic MDs (Supplementary Data Sheet S1).

Using commercially available programs, such as SureCall (Agilent Technologies, Santa Clara, CA, United States) and wANNOVAR (wannovar.wglab.org/), variant calling and annotation were carried out, respectively. As described in previous studies by our group (Nogueira et al., 2019), we filtered the variants considering the following criteria: i) the type of mutation, ii) *in silico* predictions from tools such as SIFT, MutationTaster, PolyPhen-2, and CADD (Kumar et al., 2009; Schwarz et al., 2010; Adzhubei et al., 2013; Kircher et al., 2014), iii) their presence in various databases (gnomAD, ExAC, 1000 Genomes, ClinVar, dbSNP, OMIM, and HGMD Professional), and iv) the population frequency. Variants in the Exome Variant Server databases (http://evs.gs.washington.edu) and 1000 Genomes Project (http://www.1000genomes.org) were filtered out if their minor allele frequency (MAF) was greater than 1%.

##### 2.2.2.2 Whole human mitochondrial genome

The whole human mtDNA was enriched through a single amplicon using a long-range PCR approach (Zhang et al., 2012), and libraries were prepared using Illumina’s manufacturer’s instructions. FASTQ files were aligned using the tool SeqMan NGen (DNAStar) to the mtDNA reference sequence, and commercially available programs SeqMan Pro and SeqMan NGen (DNAStar) were employed to perform the variant calling and respective annotation. Variants were filtered, considering the following criteria: i) population frequency, ii) type of mutation, iii) presence in databases (MITOMAP, MitImpact2, and HmtVar) and their *in silico* predictions, and iv) heteroplasmy exceeding 5%, as detailed in our previous study (Nogueira et al., 2019).

#### 2.2.3 Confirmatory Sanger sequencing analysis

Sanger sequencing was performed to confirm all the identified variants with the potential to be disease-causing. For this, the BigDye Terminator Cycle Sequencing Version 3.1 protocol (Applied Biosystems, Foster City, CA, United States) was used according to the standard procedures, and the sequencing results were analyzed using an ABI 3130XL DNA analyzer. Co-segregation studies were conducted in cases where DNA from additional family members was accessible to further assess the association of the identified variants with the observed phenotype.

#### 2.2.4 RNA studies

SuperScript III First-Strand Synthesis SuperMix for RT-PCR (Invitrogen, Thermo Fisher, Waltham, Massachusetts, United States) was used to synthesize cDNA, following the manufacturer’s guidelines. *NARS2* and *DARS2* cDNA were amplified using specific forward/reverse primers (Supplementary Table S1) and the following PCR program: initial denaturation at 95°C for 10 min; denaturation at 95°C for 30 s, annealing for 45 s; extension at 72°C for 60 s (35 cycles); and final extension at 72°C for 10 min.

## 3 Results

### 3.1 Demographics and clinical and genetic characteristics

**FIGURE 1:**
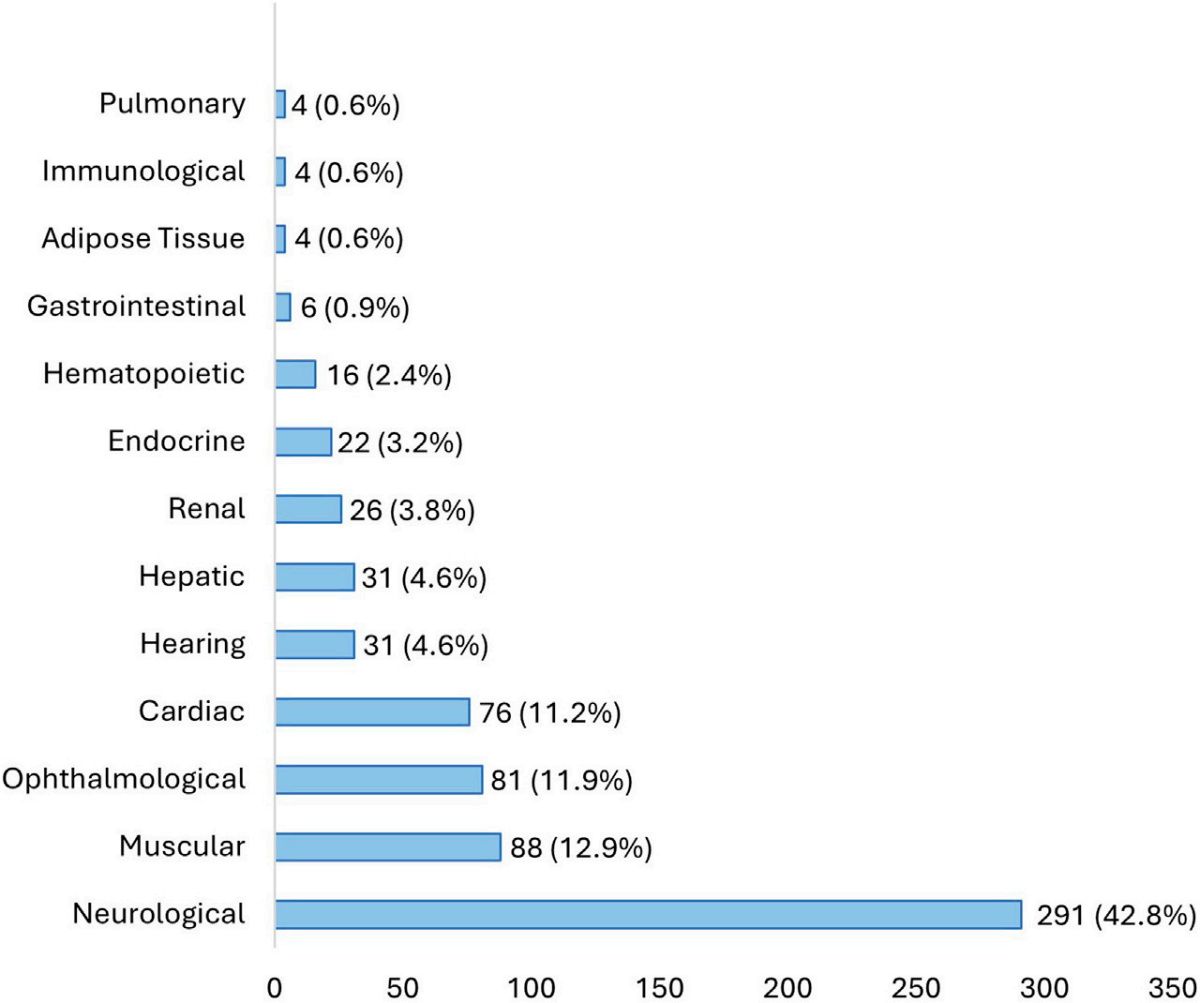
Affected systems at disease onset in our cohort.

This multicenter study enrolled patients from seven Portuguese hospitals distributed all over the country, with the majority from hospitals in the north and south. The age of onset was highly variable, ranging from 4 days to 78 years old. More than 40% of the patients presented neurologic symptoms, followed by muscular, ophthalmological, cardiac, and hearing symptoms (Figure 1).

The pediatric group represents most of the patients in this cohort, at 58% (262/450), while the adult-onset group accounted for 42% of the patients (188/450), with 29 deceased patients (6%), 23 and 6 in each group, respectively. In the post-mortem studies, a molecular diagnosis was achieved in 62% of them.

In this study, we identified a possible molecular cause in 134/450 (30%) of the studied patients, with 66% of them being identified in the nuclear genome and 34% in the mitochondrial genome.

In these positive cases, 89 patients presented pathogenic or likely pathogenic variants, more commonly in nDNA. Furthermore, we identified 45/134 patients with VUS according to the American College of Medical Genetics and Genomics guidelines (ACMG), some of which were previously published by our group (Nogueira et al., 2019).

Reflecting changes in diagnostic procedures over the last decades, among these 134 MD patients with a positive molecular result, only 39 had also undergone a muscle biopsy. Histological analysis revealed morphological abnormalities in the majority of the adults. Nineteen out of these 39 patients were also confirmed to have OXPHOS enzyme deficiencies, such as isolated defects in complex I (n = 2), complex II + III (n = 2), complex IV (n = 2), multiple MRC defects (n = 7), multiple mtDNA deletions (n = 3), and mtDNA depletion (n = 3).

### 3.2 Nuclear genome

#### 3.2.1 Pathogenic and likely pathogenic variants

Out of the 450 patients, 51 (11%) with pathogenic or likely pathogenic variants in nDNA presented 58 published and 14 novel likely pathogenic variants (Supplementary Table S2). These variants are distributed by genes involved in i) OXPHOS nuclear-encoded genes, ii) OXPHOS assembly genes, iii) mtDNA maintenance genes, iv) mtDNA transcription and translation genes, v) mitochondrial dynamics and homeostasis, vi) metabolism of substrates and cofactors, vii) metabolism of toxic compounds, and viii) other genes that mimic MDs and are known to cause monogenic disorders.

**FIGURE 2:**
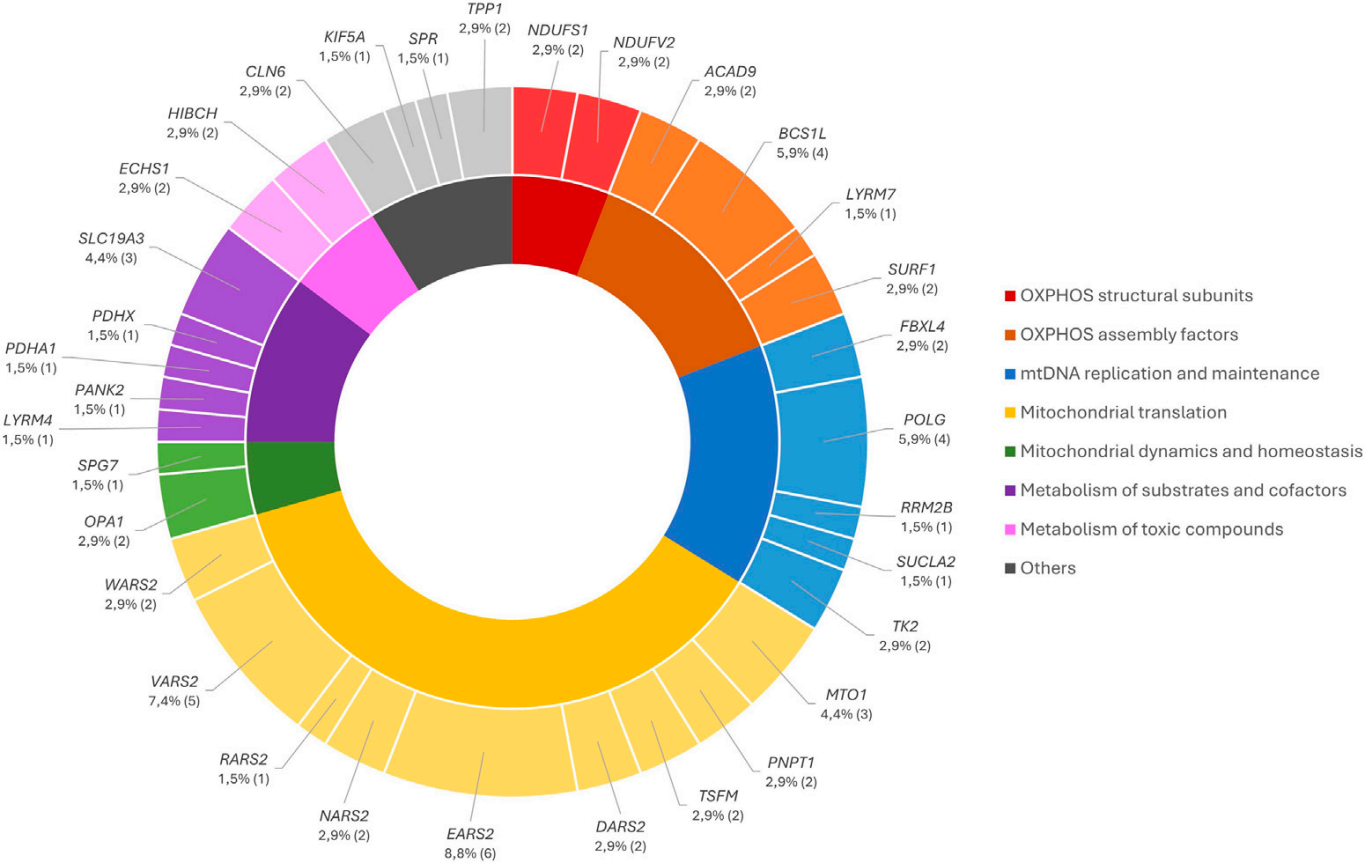
Distribution of the nDNA pathogenic/probably pathogenic variants by the affected genes according to their functional groups.

Affected genes with nDNA pathogenic and probably pathogenic variants in our cohort are described in Figure 2, according to the groups previously defined. The number of variants identified in each gene ranges from approximately 1% to 5%; however, *EARS2* and *VARS2* presented the largest number of variants, accounting for 9% and 7%, respectively.

The identified variants were comprised of i) already known causative mutations, ii) novel predicted pathogenic variants, and iii) variants that were previously validated (Nogueira et al., 2019).

Novel variants were classified as likely pathogenic when several of the following criteria were found: i) they occurred in genes where a reported pathogenic variant had already been identified, ii) the patient’s phenotypes overlapped with clinical presentations previously documented for the corresponding gene, iii) the variant exhibited an overall MAF of less than 0.01% in the gnomAD database, iv) referred *in silico* predictors labeled the variant as damaging, such as CADD stringent scores (≥20) or extremely stringent scores (≥25), and v) confirmation was obtained by Sanger sequencing on the proband’s DNA and, when available, in the DNA of family members.

#### 3.2.2 Variants of unknown significance

We identified 52 VUS, classified according to the ACMG guidelines (Richards et al., 2015), in 37/450 (8%) additional patients (Supplementary Table S3). Thirty of these VUS have been published in the literature by our group (Nogueira et al., 2019), and the remaining 22 are novel.

**FIGURE 3:**
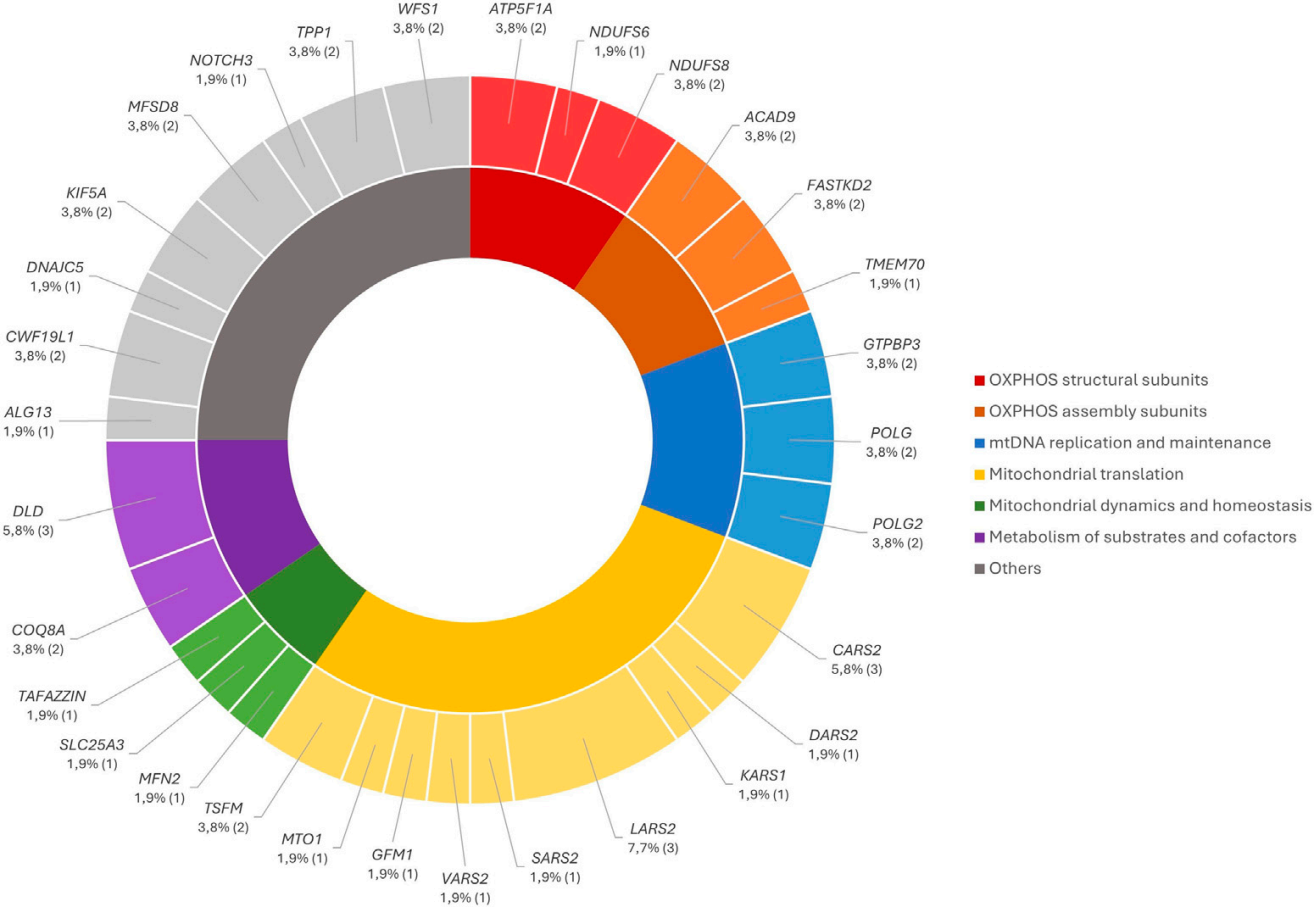
Distribution of the nDNA VUS by the affected genes according to their functional groups.

The identified VUS were distributed according to their functional groups, with the affected genes represented in Figure 3.

### 3.3 Mitochondrial genome

#### 3.3.1 Pathogenic and likely pathogenic variants

Sequencing of the whole human mitochondrial genome allowed the identification of: i) 11 additional pathogenic variants already described, ii) five likely pathogenic variants, three previously published (m.3251A>G/*MT-TL1*, m.3946G>A/*MT-ND1*, and m.6547T>C/*MT-CO1*) and two novel (m.4311G>A/*MT-TI* and m.12213G>A/*MT-TS2*), as well as iii) four single large-scale mtDNA deletions. Six of the most frequent variants of the mtDNA associated with MDs were identified in more than one patient: m.1555A>G in *MT-RNR1*, m.3243A>G and m.3271T>C in *MT-TL1*, m.8993T>G in *MT-ATP6*, m.11778A>G in *MT-ND4*, and m.13513A>G in the *MT-ND5* gene. The deafness-causing variant m.1555A>G was identified in three patients. Mitochondrial encephalopathy, lactic acidosis, and stroke-like episodes (MELAS) variants m.3243A>G and m.3271T>C were found in 11 and three patients, respectively. Leber hereditary optic neuropathy (LHON)-associated variant m.11778A>G was found in two cases, and m.8993T>G and m.13513A>G, usually associated with LS (Leigh syndrome), were present in two and three patients, respectively. The variants m.5703G>A/*MT-TN*, m.7471insC/*MT-TS1*, m.8344A>G/*MT-TK*, m.9176T>G/*MT-ATP6*, and m.10197G>A/*MT-ND3* were identified only in one patient in the studied cohort. Additionally, we were able to detect four distinct single large-scale mtDNA deletions ranging from 4 to 10 kilobases in P123 to P126.

**FIGURE 4:**
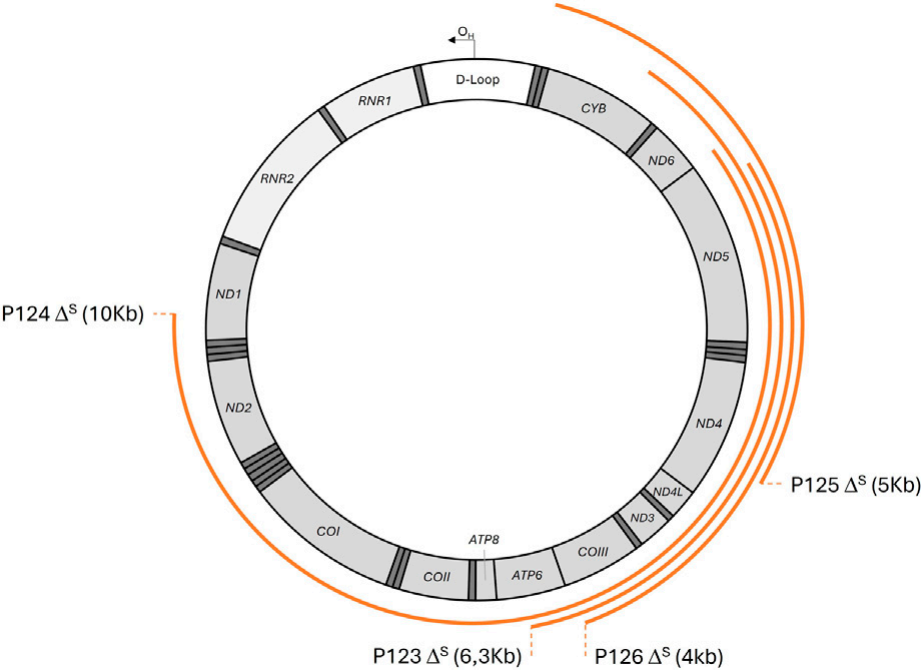
Localization and size of mtDNA large-scale deletions identified in this study.

The total variants and deletions were found in 38/450 patients (9%) (Supplementary Table S4) and were distributed in the mtDNA genes encoding the structural subunits of OXPHOS, tRNAs, and rRNAs. We highlight that the deletions, because of their large scales, encompass several genes, as shown in Figure 4.

**FIGURE 5:**
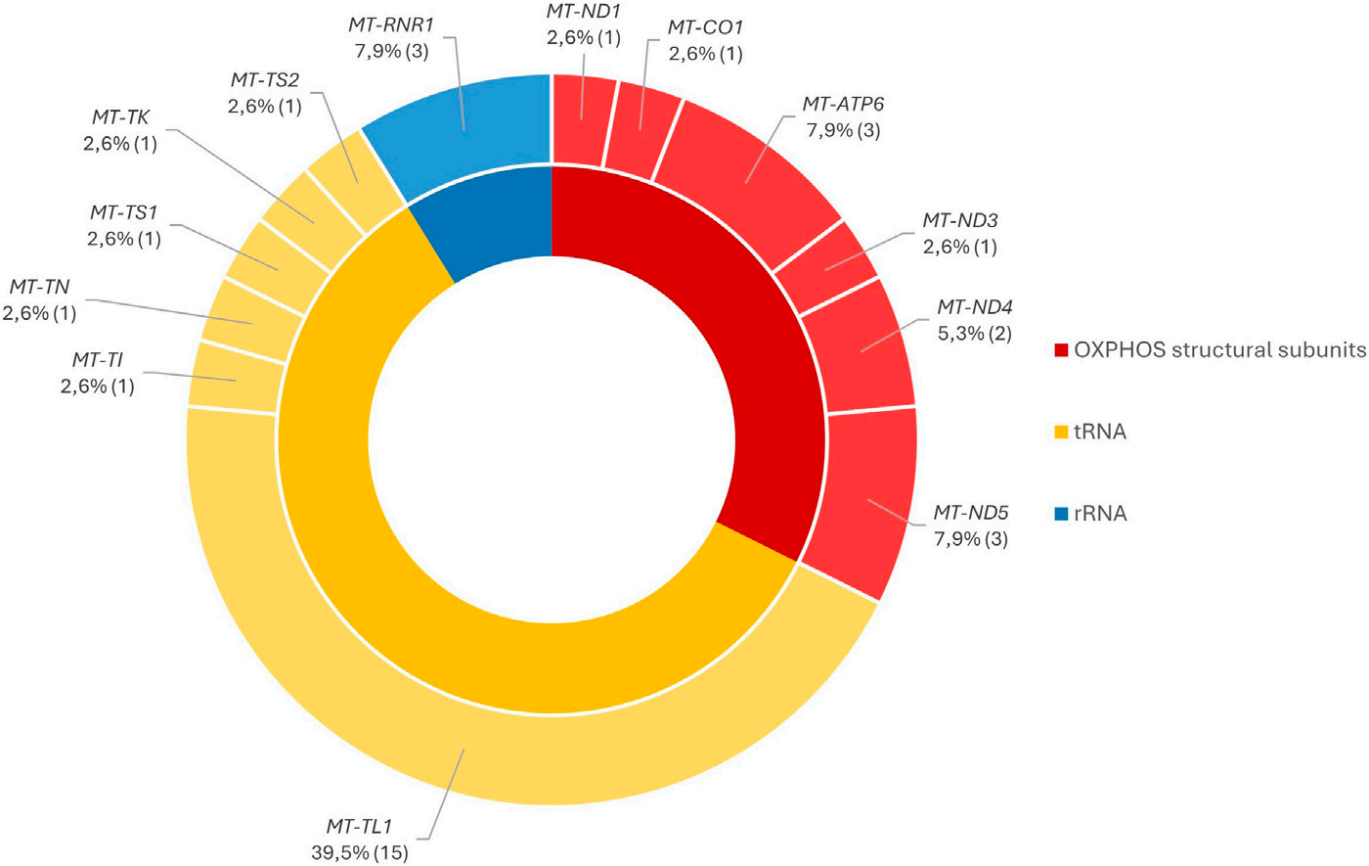
Distribution of the pathogenic/likely pathogenic variants through the mtDNA genes according to their functional groups.

Genes encoding tRNAs represent the larger group with identified variants since the *MT-TL1* gene holds the largest number of molecular characterizations in mtDNA, particularly due to the presence of m.3243A>G in 11 patients. The variants identified in the genes encoding OXPHOS subunits were, in general, equally distributed in most of them. In the case of *MT-ATP6*, *MT-ND5*, and *MT-RNR1* gene encoding the 12S rRNA, we observed a slightly higher percentage since variants in these *loci* were identified in more than one patient (Figure 5).

Most variants were detected in various degrees of heteroplasmy (ranging from 5% to 95%), except for m.1555A>G, m.9176T>G, m.10197T>G, and m.11778A>G, which were identified in homoplasmy (Supplementary Table S4). DNA extracted from muscle was only available for nine patients, harboring the following variants: m.3243A>G (P92), m.3251A>G (P103), m.3271T>C (P104), m.4311A>G (P108), m.5703G>A (P109), m.6547T>C (P110), m.10197G>A (P116), m.12213G>A (P119), and m.13513A>G (P120). A sample from buccal mucosa was studied for P93, in whom the m.3243A>G variant was also detected in this tissue. The segregation study was possible in the mothers of patients P104, P106, and P108, in whom the variants were identified in heteroplasmy, and in the mothers of patients P92, P107, P115, P119, and P120, in whom the variants were not detected.

#### 3.3.2 Variants of unknown significance in the mitochondrial genome

**FIGURE 6:**
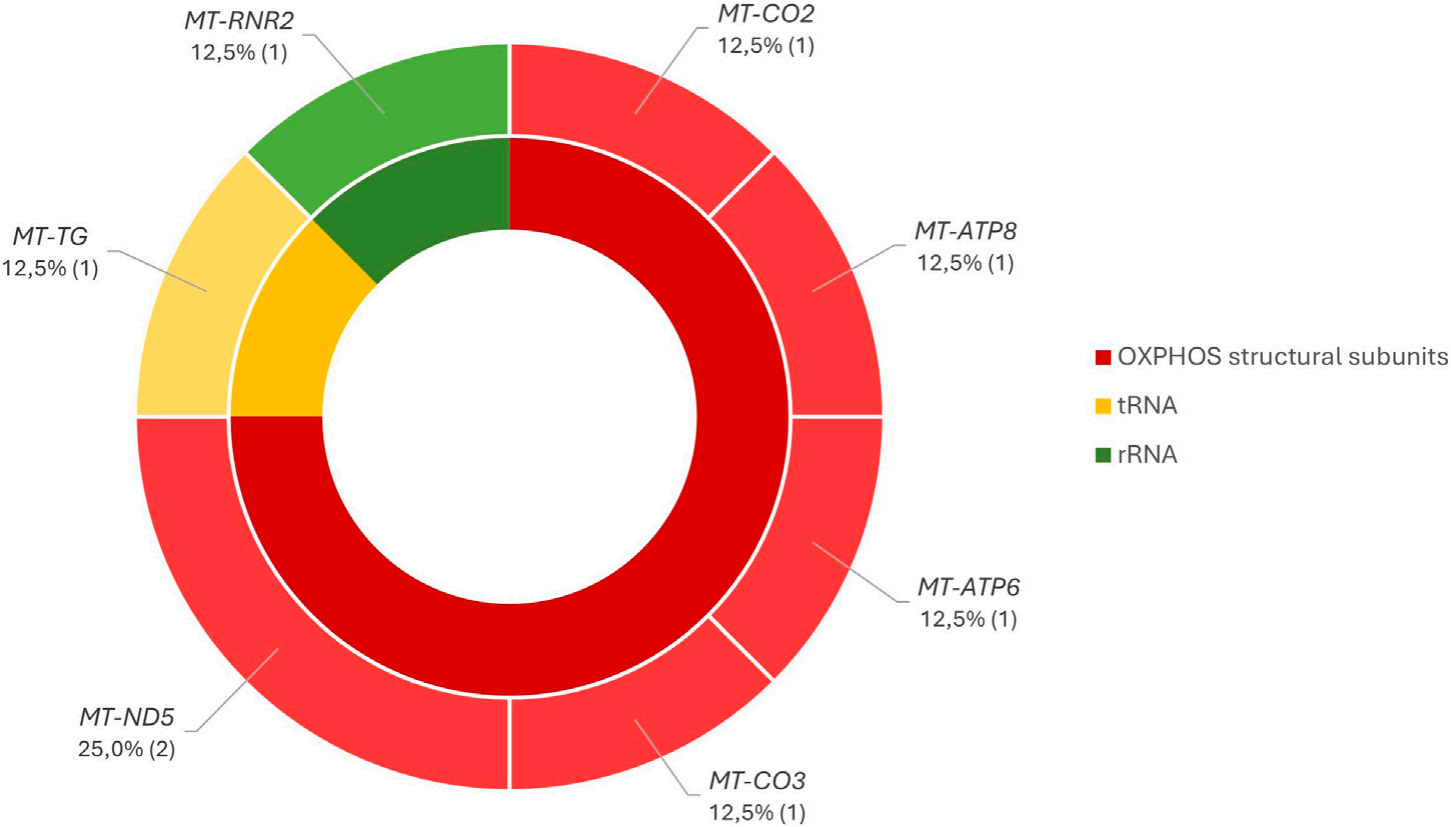
Distribution of VUS through the mtDNA genes according to their groups.

Complete sequencing of the mtDNA also revealed eight VUS (Supplementary Table S5) in eight different individuals [8/450 patients (2%)], distributed randomly in the structural genes of OXPHOS and in the genes encoding the MT-TG and MT-RNR2 (Figure 6).

All the variants were found in the heteroplasmic state, except m.9331T>C in *MT-CO3*, which was detected in homoplasmy in patient P131 and his mother. Another segregation study was possible in patient P130, the variant m.8975T>C was in heteroplasmy in the DNA from the patient’s blood and muscle and absent in his mother. In patient P134, the muscle and buccal mucosa samples were also available, but the m.13115T>C variant was not detected in those tissues.

### 3.4 Mortality rate

**FIGURE 7:**
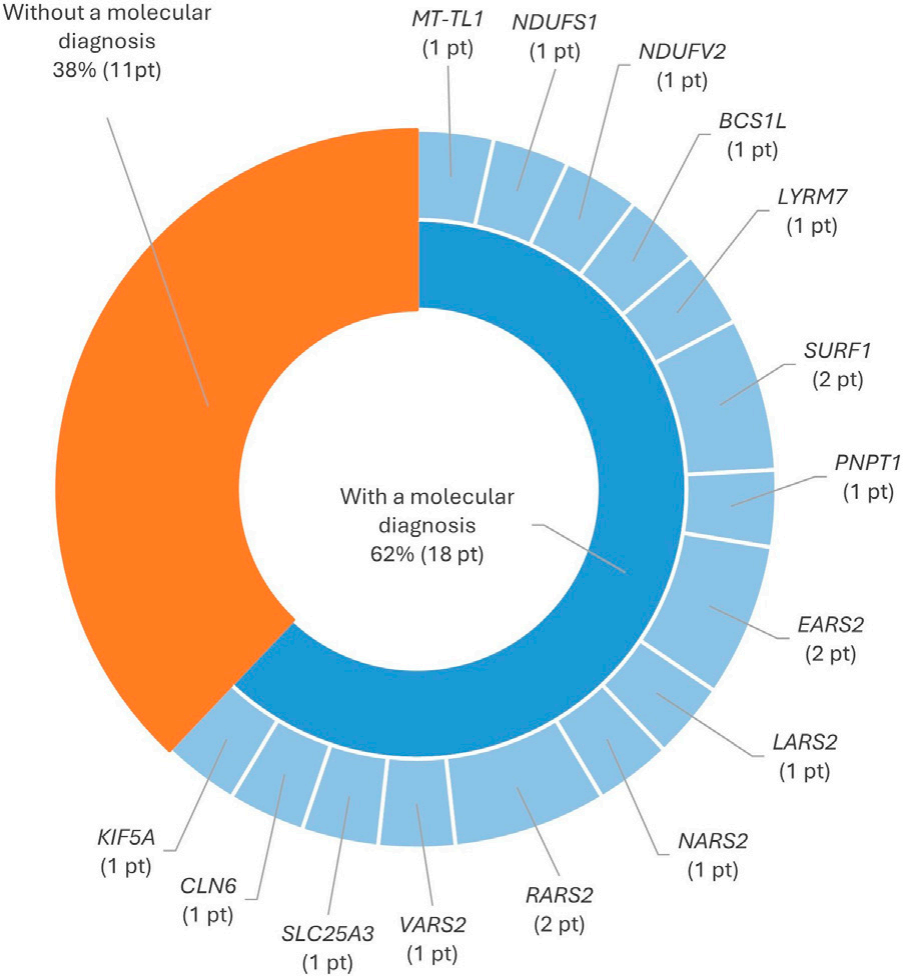
Molecular results of deceased patients.

The mortality rate in our cohort was 6% (29/450), and a molecular diagnosis was achieved in 62% of the deceased patients in this cohort. A total of 27 variants were identified: 19 pathogenic variants already described in the literature, four likely pathogenic variants, and four VUS. Most of the patients that passed away were children and presented variants in the nuclear genes involved in mitochondrial translation, as shown in Figure 7.

## 4 Discussion

Until 2016, traditional Sanger sequencing was performed to detect the most common mtDNA variants and a group of nuclear genes associated with a clinical presentation and/or a specific biochemical defect. Afterward, we applied a combined NGS approach based on a customized targeted mitochondrial panel of 213 nuclear genes, followed by the entire mitochondrial genome analysis. Initially, we applied this strategy to a cohort of 146 patients suspected of MDs without a molecular diagnosis, as detailed in our previous publication (Nogueira et al., 2019). Later, we expanded this approach to include an additional 304 patients, resulting in a comprehensive study covering a total of 450 patients. This expanded study allowed us to achieve a possible genetic diagnosis in 134/450 patients (30%), 88 in nDNA and 46 in mtDNA, identifying a total of 154 variants: 126 (82%) in the nuclear genome and 28 (18%) in the mitochondrial genome. Furthermore, 20 of these variants are novel, 72 are described, and the remaining 62 are VUS. In our study, mtDNA variants account for more adult-onset MDs, while nDNA variants more frequently result in infancy-onset MDs, which was also demonstrated in the literature in some reviews of MDs (Pronicka et al., 2016; Subathra et al., 2016).

**FIGURE 8:**
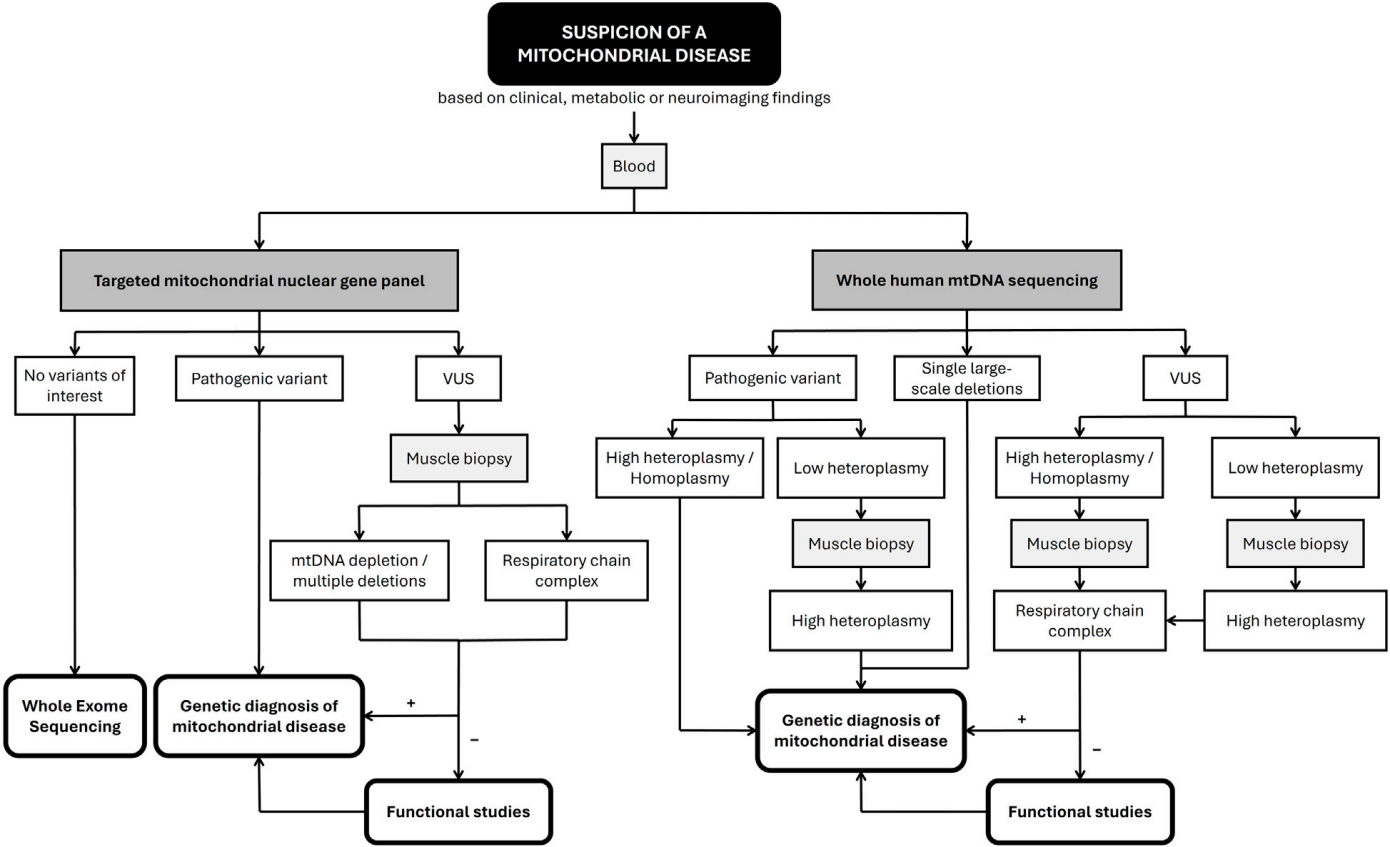
Diagnostic algorithm for a suspicion of mitochondrial disease.

Based on these results, we propose the diagnostic algorithm for MDs referred to in Figure 8.

We used blood as the primary tissue of choice for the sequence-targeted mitochondrial nuclear gene panel and the whole mitochondrial genome. However, to infer the potential pathogenicity of a VUS, detected either in nDNA or mtDNA, muscle tissue was required to perform the biochemical study of MRC, multiple deletions, and/or mtDNA depletions because it contains a large number of mitochondria. Muscle tissue was also required to detect the degree of heteroplasmy when a low level of mutated mtDNA was identified in the blood as mutation levels can change over time and increase in post-mitotic tissues (Lawless et al., 2020). Despite being a mitochondria-rich tissue, the invasive nature of muscle biopsies positions this analysis as a final step of our investigations. Furthermore, segregation studies should always be carried out for both confirmed pathogenic variants and VUS.

### 4.1 Nuclear genome

#### 4.1.1 Pathogenic and likely pathogenic variants

In 51/450 patients (11%), 12 novel likely pathogenic variants and 55 pathogenic variants, already reported in the literature, were identified in the nDNA, which were already reported in the literature (Supplementary Table S2). These variants were distributed according to the groups of genes defined in Figure 2, with most of the pathogenic variants described in the group of OXPHOS nuclear/assembly-encoded genes and the novel variants likely pathogenic in the group of mtDNA transcription and translation genes. However, we highlighted the nuclear gene *EARS2* as it had the largest number of described and novel variants identified in the pediatric group of this cohort (8%).

The likely pathogenic variants were identified in the following genes: *BCS1L* (P5), *FBXL*4 (P11), *POLG* (P14 and P15), *RRM2B* (P16), *MTO1* (P19), *TSFM* (P22 and P23), *EARS2* (P28), *NARS2* (P29), *RARS2* (P30 and P31), *VARS2* (P32 and P34), and *SLC19A3* (P45). Among this group, we highlight P29, who was a female newborn with intrauterine growth restriction and preterm birth. After birth, she developed severe mitochondrial dysfunction, presenting lactic acidosis, severe generalized hyperaminoaciduria, congenital heart disease, neonatal diabetes, and omphalocele. Her clinical condition had a progressively fatal outcome. The primary analysis of P29 identified a described pathogenic variant in *NARS2*, c.500A>G (p.His167Arg), in heterozygosity. This gene is responsible for encoding the enzyme asparaginyl-tRNA synthetase, which is imported into the mitochondria in order to catalyze the binding of asparagine to the respective tRNA molecule (Simon et al., 2015). Biallelic pathogenic variants in this gene are one of the causes of OXPHOS complex dysfunction and result in neurodegenerative disorders, such as spastic paraplegia, Leigh syndrome, and Alpers syndrome (Štěrbová et al., 2021). As it is an autosomal recessive disease and only one causal allele had been identified, we continued the study of *NARS2* cDNA, which revealed a large heterozygous deletion covering exons 7, 8, and 9 [c.690-?_959+?del (p.Ala231_Ile320del)], confirming the definitive diagnosis of this patient.

The following pairs of patients, P9 and P10, P12 and P13, P22 and P23, P25 and P26, and P30 and P31, are siblings harboring pathogenic and/or likely pathogenic variants in the *SURF1*, *FBXL4*, *TSFM*, *EARS2*, and *RARS2* genes, respectively. The clinical phenotypes of the siblings with pathogenic variants are similar to those described in the literature (Tiranti et al., 1998; Steenweg et al., 2012).

Most of the characterized patients with pathogenic and likely pathogenic variants in the nuclear genome were children (76%), which is in agreement with the literature (Chinnery et al., 2014). The remaining 23% of patients were adults and presented variants in the following genes: *POLG*, *RRM2B*, *OPA1*, *KIF5A*, *SPR*, *NDUFV2*, *TK2*, *PNPT1*, *VARS2*, *WARS2*, *PDHX*, and *SPG7*, the first five with autosomal dominant patterns and the remaining in autosomal recessive patterns. Moreover, multiple mtDNA deletions were confirmed in two of the patients with variants in the *POLG* and *TK2* genes, consistent with what is described in the literature.

#### 4.1.2 Variants of unknown significance

Furthermore, we identified 54 VUS (Supplementary Table S3), according to the ACMG, in 37/450 patients (8%), which were distributed according to their functional groups defined in Figure 3.

In the group of OXPHOS subunits and their respective assembly factors 10 VUS were identified, seven of which were already described by our group in previous studies (Nogueira et al., 2019), and three were novel; one was identified in *NDUFS1* and the others in *ATP5F1A*.

In the mtDNA replication and maintenance group, a total of six VUS were detected, with three of them identified for the first time in this study in the *GTPBP3* and *POLG2* genes.

**FIGURE 9:**
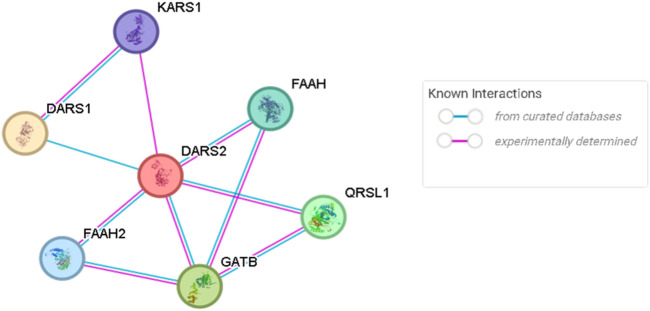
Physical network of genetic interactions associated with the *DARS2* gene. Adapted from the STRING platform (https://string-db.org/).

The group of mitochondrial DNA transcription and translation genes group accounts for 15 VUS, nine of which were novel, localized in the *CARS2*, *KARS1*, *LARS2*, *VARS2*, *TSFM*, and *GFM1* genes. In this group, we highlight P65, a 2-year-old male child with hyperlactacidemia, hyperalaninemia, ataxia, non-progressive hypotonia, and a normal MRI. His primary molecular analysis identified a described heterozygous variant in the *DARS2*, c.90C>A (p.Tyr30*), but the cDNA study did not allow the identification of the second causal allele in this gene. Thus, as it is described in the literature (Papadimitriou et al., 2019), there are additional genes (*KARS1*, *DARS1*, *FAAH*, *FAAH2*, *GATB*, and *QRSL1*) that interact with *DARS2*, as shown by the STRING platform (https://string-db.org/) (Figure 9).

Among these, *KARS1* (OMIM *601421) stands out, in which the variant c.1609C>T (p.Arg537Trp) was identified in heterozygosity. In addition to these two proteins having some sequence similarity, their interaction has already been previously described in the literature and may reinforce this hypothesis (Robinson et al., 2000).

Co-segregation studies were also performed, with his father being the carrier of the *KARS1* variant and his mother being the carrier of the *DARS2* variant, confirming the digenic inheritance in this patient. In the group of dynamics and homeostasis, three VUS were identified; one of them was novel in the *MFN2* gene and present in two siblings (P73 and P74) with a similar clinical picture of neurodegenerative disease. In the metabolism of substrates and cofactors group, five VUS were identified, three of them novel, in *COQ8A* and *DLD* genes. Finally, in the group of genes that mimic MDs, a total of 13 VUS were identified, with six of them being novel in *ALG13*, *CWF19L1*, *DNAJC5*, *KIF5A*, and *NOTCH3* genes.

The identified VUS require additional analysis through animal and/or functional studies to establish their pathogenicity. Although it is beyond the scope of this work, these studies may involve various approaches, including the search for biomarkers, rescue experiments, animal models, micro-organism models, CRISPR/Cas9 technology, induced pluripotent stem cells (iPSC), and other relevant methodologies. To definitively validate the impact of these variants on protein function, these studies are essential for conclusive diagnosis within this cohort of patients.

### 4.2 Mitochondrial genome

The sequencing of mtDNA revealed a total of 24 variants (16 pathogenic/likely pathogenic and 8 VUS) and four single large-scale mtDNA deletions, which eventually led to a molecular diagnosis in 46/450 (10%) patients. The percentage of variants identified in the mitochondrial genome was conditioned by the fact that some of the patients with a well-defined syndrome were ruled out since the first approach of their study was orientated and positive for the most frequent variants in mtDNA or multiple deletions in the muscle biopsy. Nevertheless, we still identified some of these frequent variants in the patients of this cohort since the approach has been to sequence the complete mtDNA by NGS in all suspected MD patients. Another factor for the relatively low percentage of identified variants in mtDNA was the age of the patients in this cohort, in which most of the patients were pediatric, 262 (58%), for whom the presence of a causal variant in the nuclear genes associated with MDs is more probable.

#### 4.2.1 Pathogenic/likely pathogenic variants

The associated phenotypes with the eleven previously reported pathogenic variants in mtDNA found in this research were, generally, in agreement with the already described presentations. However, we identified the deafness-associated m.1555A>G/*MT-TRNR1* variant in patient P89, who presented parkinsonism without hearing loss, and in patients P90 and P91, who had neurological symptoms in addition to deafness. The association of the m.1555A>G variant with distinct presentations is already known, and recently, it has been hypothesized that this variant may be associated with multi-organ disorder, with penetrance correlated with the level of heteroplasmy in different tissues (Sobenin et al., 2013; Valiente-Pallejà et al., 2018; Finsterer, 2020). Another variant that is usually associated with deafness, the m.7471insC in *MT-TS1*, was identified in patient P111, who presented with progressive external ophthalmoplegia, head drop syndrome, and tetraparesis. This variant was formerly reported in patients with neurological presentations (Jaksch et al., 1998; Cardaioli et al., 2006; Valente et al., 2009).

It is common for an mtDNA variant to cause a broad spectrum of clinical symptoms at variable heteroplasmy levels, and this was observed for the MELAS m.3243A>G in *MT-TL1*, which was identified in patients with variable but known presentations (Vilarinho et al., 1997; Vilarinho et al., 1999). We confirmed that the level of heteroplasmy in the blood is not representative of the penetrance of this mutation, and, although it was not always possible in this study, the load of m.3243A>G should be estimated in other tissues (Chin et al., 2014; Shen and Du, 2021). This evaluation would be particularly relevant for patients P92 and P95, who must have a higher rate of mutation in the heart and kidney tissues, respectively.

It is also relevant to mention that in patient P109, in addition to the *MT-TN* variant m.5703G>A, multiple mtDNA deletions were identified in the muscular biopsy. The m.5703G>A variant has been reported with a similar presentation, but multiple mtDNA deletions were not reported (Zereg et al., 2020). We speculate that the presence of this variant may have enhanced the level of multiple mtDNA deletions in this patient and/or that these rearrangements were a consequence of aging despite her age of 57 years (Sevini et al., 2014).

We also highlight our group report about the m.9176T>G in P115, in which the low citrulline level prompted the investigation of mtDNA and revealed this *de novo* pathogenic variant in a nearly homoplasmic state (Baldo et al., 2023).

The likely pathogenic variants m.3251A>G/*MT-TL1* in patient P103, m.3946G>A/*MT-ND1* in patient P107, and m.6547T>C/*MT-CO1* in patient P110 have previously been described but have not been classified with a confirmed pathogenic status. The m.3251A>G variant has already been associated with myopathy, and, in our patient, the biochemical study of the complexes of MRC revealed severe multiple deficiencies, which are usually present in causal mutations in mitochondrial tRNA genes (Sweeney et al., 1993; Houshmand et al., 1996). Additionally, the percentage of heteroplasmy in the muscle biopsy was higher (95%) than the rate detected in the blood (70%). The m.3946G>A variant appeared *de novo* in LS patient P107. There is a previous report with the same presentation, and the variant was detected in a high level of heteroplasmy in his blood sample. These findings, the *in silico* predictions, the frequency of 0.002% in the MITOMAP database, and the conservation of this *locus* are in favor of a pathogenic status (Ogawa et al., 2017). The m.6547T>C variant identified in P110, first described by Yoneda in 1990 and later reported by our group (Pereira et al., 2019), was recently reclassified by the new version of APOGEE (APG2) as likely pathogenic.

The m.4311G>A/*MT-TI* variant in patient P108 and m.12213G>A/*MT-TS2* in patient P119 are newly reported in our study as being associated with diseases. The m.4311G>A variant exhibited high heteroplasmy in the blood and muscle samples of this patient and was present in only 15% of the molecules in the blood sample of the asymptomatic mother. The MitoTip tool classified this variant as likely pathogenic; it is absent from the population database of MITOMAP, and it is in a conserved position in the mtDNA. The m.12213G>A/*MT-TS2* variant was detected with a 25% heteroplasmy rate in the blood sample and 98% in the muscle biopsy of patient P119 and was absent in her asymptomatic mother. The MitoTip tool classified the variant as possibly pathogenic; it is absent from the MITOMAP database, and it is a highly conserved *locus* in mtDNA. Additionally, the MRC study revealed multiple deficiencies of the MRC complexes.

We detected four single large-scale mtDNA deletions in the blood samples of four pediatric patients. These deletions are generally not detected in the blood, except for Pearson syndrome (PS)/ Kearn–Sayre syndrome (KSS). It is worth noting that these four patients (P123–P126) did not exhibit the classical presentation of PS, and their mitochondrial genome were screened due to a suspected MD with no indication of a particular syndrome. These cases bring new insights for the clinical presentation of PS and add an advantage for the utility of NGS analysis of mtDNA in blood samples of children with a suspected MD (Wild et al., 2020; Yoshimi et al., 2022).

#### 4.2.2 Variants of unknown significance

In accordance with the recommendations of the ACMG/AMP (Association of Molecular Pathology) and guidelines for mitochondrial DNA variant interpretation, we classified eight of the variants found in the mtDNA as VUS (McCormick et al., 2020).

The frequency of all these VUS is below 0.002% in the MITOMAP database, and these variants have been detected in heteroplasmy, apart from m.9331T>C in *MT-CO3*. This variant found in patient P131, although in homoplasmy, was also identified in his mother, who presented cardiac symptoms and myalgia like her son. Three of the VUS, m.2862C>T/*MT-RNR2*, m.10011A>G/*MT-TG*, and m.13115T>C/*MT-ND5*, are newly reported to be associated with diseases in this study. The remaining variants have already been described, and we emphasize that the m.8382C>T/*MT-ATP8* and m.8975T>C/*MT-ATP6* variants were investigated by functional studies (Rucheton et al., 2020). We also highlight that the m.8975T>C variant in *MT-ATP6* appeared *de novo* in patient P130 with no family history of MDs. The m.12425delA variant, located in *MT-ND5,* a hotspot gene for mtDNA pathogenic variants, predicted a severe truncation of the mature ND5 protein and was reported in a young girl with an isolated complex-I deficiency associated with an unusual MD presentation (Alston et al., 2010). Multiple lines of computational evidence support a deleterious effect for most of the VUS, except for the m.2862C>T in *MT-RNR2* variants since there are currently no readily accessible informatics tools for the prediction of rRNA variant effects.

As mentioned above, there is some evidence suggesting that these variants may play a causative role in the clinical presentation of these patients with VUS. However, further studies must be performed involving segregation investigation, the rating of heteroplasmy of the variants in other tissues, the determination of the activity of the complexes of MRC, and functional studies.

### 4.3 Mortality rate

The mortality rate in our cohort (6%) was below the range of 14%–46% described in several other studies looking into MD mortality (Debray et al., 2007; Eom et al., 2017; Papadopoulos et al., 2020; Wong et al., 2023). Of the 62% deceased patients with a molecular diagnosis, the majority were children and presented mutations in the nuclear genes involved in mitochondrial translation. Defects in nucleus-encoded mitochondrial aminoacyl-tRNA synthetases, key components of the mitochondrial translation apparatus, result in severe combined MRC deficiencies and often lead to a fatal outcome in infancy (Gomez and Ibba, 2020).

## 5 Conclusion

The successful completion of the Human Genome Project in 2003, coupled with significant advancements in NGS technologies, marked unparalleled advances in the field of human genomics research, which has reshaped clinical medicine and biomedical exploration.

This research provided a comprehensive overview of the genetic makeup and clinical features of a group of Portuguese individuals suspected of having MDs. The NGS data provided by our study emphasize the effectiveness of employing this combined NGS approach for achieving genetic diagnoses in numerous cases involving both children and adults. Many of these cases have proven challenging to resolve through conventional Sanger sequencing-based screening of specific nuclear genes and the most common mtDNA mutations, which is why the use of custom-designed panels is so useful when seeking to obtain a molecular diagnosis in a timely and cost-effective manner. Additionally, concerning *post-mortem* diagnoses, we emphasize the significance of NGS data, which can facilitate subsequent prenatal diagnosis.

Comparing this study with our previous study (Nogueira et al., 2019), for this extended cohort of studied patients, there was a significant contribution of this manuscript to achieve more molecular diagnosis, increasing the possible genetic diagnosis from 25% to 30%. Nevertheless, to address the remaining 316 patients (70%) without a definitive genetic diagnosis, performing whole-exome sequencing analysis could increase the molecular characterization for this set of patients, as the analysis of targeted gene panels often leads to the omission of newly discovered genetic causes.

Nonetheless, as a national laboratory for the study and research of MDs, the results of our center contributed to the expansion of the mutational spectrum of pathogenic variants and are expected to provide appropriate family counseling, including prenatal and preimplantation screening. Given the genetic heterogeneity of these disorders and the absence of effective therapeutic options to date, these results carry substantial implications for enhancing our understanding and management of MDs.

## Data Availability

The original contributions presented in the study are included in the article/Supplementary Material; further inquiries can be directed to the corresponding author.
