# Supplementary material for: The genetic landscape of mitochondrial diseases in the next-generation sequencing era: a Portuguese cohort study
Source: Front Cell Dev Biol. 2024 Feb 23;12:1331351. doi: 10.3389/fcell.2024.1331351 (PMC10920333; doi:10.3389/fcell.2024.1331351)
Supplement: Supplementary file 1 [file Table1.docx]

| Gene  (cDNA) | Sequence 5’ 🡪 3’ | Melting Temperature | Fragment size |
| --- | --- | --- | --- |
| *NARS2* | F1: GGGCTCTGGAGTGCCTTAG | 60.0 ºC | 616 bp |
|  | R1: CGCTTCACTGCGAATCCT |  |  |
|  | F2: GCAGATTCAGGCCTTGACAG |  | 526 bp |
|  | R2: GCCTCCGGCTCTGAGAAT |  |  |
|  | F3: TGACTCTGAGGGAGCTGGA |  | 525 bp |
|  | R3: TTGCCACAGTGCTTCACC |  |  |
|  | F4: AAGCATCCCAGAACTTCACC |  | 646 bp |
|  | R4: TTCTTGCGGGAGCTCATC |  |  |
| *DARS2* | F1: GTGCGGAGGAGGCCTTA | 60.0 ºC | 614 bp |
|  | R1: CAAAGGGCAGCTTCTTGC |  |  |
|  | F2: ATGTGAAGCCCCTGTGGA |  | 605 bp |
|  | R2: TGTCATTGGGCCAGGAAT |  |  |
|  | F3: TTCAGGTTGCCCGATGTT |  | 724 bp |
|  | R3: GCCGATTCCAGCTCTCTG |  |  |
|  | F4: GAGTGGTGCTCCGTGACC |  | 668 bp |
|  | R4: TTGCCTGGATTCCTTCCA |  |  |

**Supplementary Table S1 –** cDNA primers of NARS2 and DARS2 genes.

F: Forward Primer ; R: Reverse primer ; bp: Base pairs
