# Supplementary material for: The genetic landscape of mitochondrial diseases in the next-generation sequencing era: a Portuguese cohort study
Source: Front Cell Dev Biol. 2024 Feb 23;12:1331351. doi: 10.3389/fcell.2024.1331351 (PMC10920333; doi:10.3389/fcell.2024.1331351)
Supplement: Supplementary file 2 [file Table2.docx]

| Supplementary Table S2 – Pathogenic and likely pathogenic variants identified in nuclear DNA by next generation sequencing. | | | | | | | | | | | | | | | | |
| --- | --- | --- | --- | --- | --- | --- | --- | --- | --- | --- | --- | --- | --- | --- | --- | --- |
|  | | | | | | | | | | | | | | | | |
| Patient | **Dx**  **Age** | **Gender** | **Muscular Defects** | **Clinical Presentation** | **Gene** | **Protein change** | **Nucleotide change** | **SIFT** | **PP2** | **MT** | **CADD** | **gnomAD (Overall)** | **gnomAD (Highest freq)** | **Segregation Data** | **Trait** | **References** |
| P1 | 1y ^†^ | M | NA | Delayed psychomotor development; axial hypotonia; poor weight evolution; nystagmus; MRI compatible with Leigh syndrome; hyperlactacidemia; failure to grow; post-infectious deterioration. | *NDUFS1* | p.Lys157Met/ p.Glu600Gln | c.470A>T/ c.1798G>C | D D | D D | D D | 28.7 28.9 | - - | -  - | Father: htz Mother: htz | AR | Baldo (2023)/ Baldo(2023) |
| P2 | 2y ^†^ | F | RC: N^a^ | Leigh syndrome. | *NDUFV2* | Splicing/  splicing | c.120+5_120+8delGTAA/ c.120+5_120+8delGTAA | - - | - - | - - | -  - | 3.98e^-6^ 3.98e^-6^ | 8.8e^-6^ (EUR) 8.8e^-6^ (EUR) | Father: htz Mother: htz | AR | Benit (2003)/ Benit (2003) |
| P3 | 21y | F | RC: N | Ataxia; pyramidal syndrome; alteration of left ventricular repolarization; high lactate and alanine. | *NDUFV2* | p.*250Glnext*34/ p.*250Glnext*34 | c.748T>C/  c.748T>C | - - | - - | N N | 22.7 22.7 | -  - | -  - | Mother: htz Father: htz | AR | Nogueira (2019)/ Nogueira (2019) |
| P4 | 11y | M | CI (18%) | Cardiomyopathy; lactic acidosis. | *ACAD9* | p.Glu413Lys/ p.Pro616Ser | c.1237G>A/ c.1846C>T | D D | D D | D D | 33 31 | 1.99e^-5^ 3.98e^-6^ | 1.23e^-4^ (AFR) 2.89e^-5^ (LAT) | Father: htz Mother: htz | AR | Nouws (2010)/ Nogueira (2019) |
| P5 | 9y ^†^ | M | NA | Mitochondrial hepatopathy; metabolic acidosis; hyperlactacidemia, intra-uterine growth restriction; cholestasis. | *BCS1L* | UTR splicing/ p.Arg224Cys | c.-147A>G/ c.670C>T | - D | - D | - D | - 35 | - 3.98e^-6^ | - 8.78e^-6^ (EUR) | NA | AR | Gil-Borlado (2009)/ This study |
| P6 | 1y | F | NA | Psychomotor regression; epilepsy; axial hypotonia with peripheral hypertonia; hyperlactacidemia. | *BCS1L* | p.Arg183Cys/ p.Arg184Cys | c.547C>T/ c.550C>T | D D | D D | A A | 34 34 | 2.83e^-5^ 1.59e^-4^ | 8.01e^-5^ (AFR) 2.8e^-3^ (AJ) | NA | AR | Fernandez-Vizarra (2007)/ Hinson (2007) |
| P7 | 2y ^†^ | F | CII+ CIII  (35%);  Depl. (70%) | Delayed psychomotor development; encephalopathy; respiratory failure; metabolic acidosis; hyperlactacidemia. | *LYRM7* | p.Asp25Asn/ p.Asp25Asn | c.73G>A/ c.73G>A | D D | D D | D D | 31 31 | - - | -  - | Father: htz Mother: htz | AR | Invernizzi (2013)/ Invernizzi (2013) |
| P8 | 5y | M | NA | Hypotonia; global developmental delay; hyperlactacidemia; metabolic acidosis. | *LYRM7* | p.Asp25Asn/ p.Asp25Asn | c.73G>A/ c.73G>A | D D | D D | D D | 31 31 | - - | -  - | NA | AR | Invernizzi (2013)/ Invernizzi (2013) |
| P9 | 4y ^†^ | F | NA | Hypotonia; global developmental delay; tremor; MRI compatible with Leigh syndrome. | *SURF1* | p.Ala13Cysfs*65/ p.Ser282Cysfs*9 | c.19_35dup/ c.845_846del | - - | - - | - - | - - | - 9.58e^-5^ | -  1.48e^-4^ (EUR) | NA | AR | Tiranti (1998)/ Tiranti (1998) |
| P10 | 2y ^†^ | F | NA | Preterm; hypotonia; global developmental delay; MRI compatible with Leigh syndrome. | *SURF1* | p.Ala13Cysfs*65/ p.Ser282Cysfs*9 | c.19_35dup/ c.845_846del | - - | - - | - - | - - | - 9.6e^-5^ | -  1.48e^-4^ (EUR) | NA | AR | Tiranti (1998)/ Tiranti (1998) |
| P11 | 7m | M | NA | Fetal growth restriction; hyperlactacidemia; hypoglycemia; generalized hypotonia; hypertrophic cardiomyopathy; bilateral lesions in the basal ganglia. | *FBXL4* | p.Gln124*/ p.Gln124* | c.370C>T/ c.370C>T | - - | - - | A A | 37 37 | 3.98e^-6^ 3.98e^-6^ | 8.79e^-6^ (EUR) 8.79e^-6^ (EUR) | Mother: htz/ Father: htz | AR | This study/ This study |
| P12 | 14y | F | CI (21%) | Neutropenia; hypotonia; epilepsy; renal tubular acidosis; delayed psychomotor development; growth retardation. | *FBXL4* | p.Cys547*/ p.Cys547* | c.1641_1642delTG/ c.1641_1642delTG | - - | - - | - - | - - | 1.24e^-4^ 1.24e^-4^ | 2.9e^-4^ (AJ) 2.9e^-4^ (AJ) | Father: htz Mother: htz | AR | Huemer (2015)/ Huemer (2015) |
| P13 | 5y | F | NA | Neutropenia; hypotonia; renal tubular acidosis; delayed psychomotor development. | *FBXL4* | p.Cys547*/ p.Cys547* | c.1641_1642delTG/ c.1641_1642delTG | - - | - - | - - | - - | 1.24e^-4^ 1.24e^-4^ | 2.9e^-4^ (AJ) 2.9e^-4^ (AJ) | Father: htz Mother: htz | AR | Huemer (2015)/ Huemer (2015) |
| P14 | 4y | M | NA | Leigh syndrome. | *POLG* | p.Thr251Ile/ p.Pro587Leu/ splicing | c.752C>T/ c.1760C>T/ c.1950-21C>T | T D - | B D - | A A - | 7.18 27.8 - | 1.54e^-3^ 1.54e^-3^ 6.32e^-3^ | 2.67e^-3^ (EUR) 2.65e^-3^ (EUR) 6.62e^-2^ (AFR) | Mother: htz Mother: htz Father: htz | AR | Lamantea (2002)/ Van Goethem (2003)/ This study |
| P15 | 45y | F | Δ^M^ | Cerebellar ataxia since age 20. | *POLG* | p.Ile948Thr | c.2843T>C | T | P | D | 21.4 | - | - | NA | AD | This study |
| P16 | 55y | F | NA | Muscle weakness; ptosis. | *RRM2B* | p.Ile47Val | c.139A>G | D | D | D | 23 | 7.07e^-6^ | 4.0e^-5^ (AFR) | NA | AD | This study |
| P17 | 9y | M | CII (27%), CIV (16%) | Neurological and muscular involvement; delayed psychomotor development; severe dystonia with tetraparesis; muscle weakness. | *SUCLA2* | p.Ala307Val/ p.Ala307Val | c.920C>T/ c.920C>T | T T | B B | D D | 18.61 18.61 | - - | - - | Father: htz Mother: htz | AR | Huang (2017)/  Huang (2017) |
| P18 | 44y | F | Δ^M^ | Ophthalmoparesis, dystonia. | *TK2* | p.Ser138Gly/ p.Ala139Thr | c.412A>G / c.415G>A | D D | D D | D D | 26.5 33 | 3.98e^-6^ 9.19e^-5^ | 5.43e^-5^ (EA) 4.8e^-4^ (AFR) | NA | AR | Nogueira (2019)/ Garone (2018) |
| P19 | 12y | M | CIV (25%) | Metabolic acidosis on day 5 of life; delayed psychomotor development; epilepsy; dilated cardiomyopathy; hyperactivity; hyperlactacidemia. | *MTO1* | p.Met138Serfs*6/ p.Arg484Trp | c.413delT/ c.1450C>T | - D | - D | - D | - 34 | 3.98e^-6^ 2.12e^-5^ | 2.89e^-5^ (LAT) 4.65e^-5^ (EUR) | NA | AR | This study/ O’Byrne (2018 |
| P20 | 8y | F | NA | Hypertrophic cardiomyopathy; hyperlactacidemia. | *MTO1* | p.Arg431Trp/ p.Arg484Trp | c.1291C>T/ c.1450C>T | T D | B D | D D | 35 34 | 7.95e^-6^ 2.12e^-5^ | 5.43e^-5^ (EA) 4.65e^-5^ (EUR) | Mother: htz Father: htz | AR | Nogueira (2019)/ O’Byrne (2018) |
| P21 | 22y ^†^ | F | RC: N | Cognitive impairment; congenital cerebellar ataxia; sensorineural hearing loss; nystagmus; cerebellar atrophy; progressive motor and sensory neuropathy; encephalomyopathy. | *PNPT1* | splicing/ p.Ala507Ser | c.1177-2A>C/ c.1519G>T | - T | - P | D D | 21.6 25.7 | - 2.13e^-4^ | - 4.57e^-4^ (EUR) | Mother: htz Father: htz | AR | Nogueira (2019)/  Slavotinek (2015) |
| P22 | 8y | F | NA | Hypertrophic cardiomyopathy; chorea. | *TSFM* | p.Cys64Tyr/ p.Cys261Ser | c.191G>A/ c.782G>C | D T | D B | D D | 29.2 23.2 | 3.19e^-5^ 4.11e^-6^ | 9.2e^-4^ (Other) 9.1e^-6^ (EUR) | Father: htz Mother: htz | AR | This study/  Emperador (2016) |
| P23 | 4y | M | NA | Hypertrophic cardiomyopathy; chorea. | *TSFM* | p.Cys64Tyr/ p.Cys261Ser | c.191G>A/ c.782G>C | D T | D B | D D | 29.2 23.2 | 3.19e^-5^ 4.11e^-6^ | 9.2e^-4^ (Other) 9.1e^-6^ (EUR) | Father: htz Mother: htz | AR | This study/  Emperador (2016) |
| P24 | 3y | F | NA | Ataxia; speech delay; generalized hypotonia; leukodystrophy; macrocephaly; motor delay; muscular hypotonia; severe global development delay. | *DARS2* | p.Ser45Gly/ splicing | c.133A>G/ c.228-21_228-20delTTinsC | D - | B - | A - | 22.8 - | -  - | -  - | Mother: htz Father: htz | AR | Scheper (2007)/ Scheper (2007) |
| P25 | 2m ^†^ | M | CI(37%), CIV (18%)^a^ | Leukoencephalopathy; feeding difficulties with poor weight progression and axial hypotonia. | *EARS2* | p.Met1?/ p.Glu96Lys | c.1A>G/ c.286G>A | D T | P P | D D | 15.75 24.9 | 3.19e^-5^ - | 6.49e^-5^ (EUR) - | Father: htz Mother: htz | AR | Steenweg (2012)/ Steenweg (2012) |
| P26 | 8y | M | CIII (39%), CIV (24%), CV (24%) | Myopathy; leukoencephalopathy. | *EARS2* | p.Met1?/ p.Glu96Lys | c.1A>G/ c.286G>A | D T | P P | D D | 15.75 24.9 | 3.19e^-5^ - | 6.49e^-5^ (EUR) - | Father: htz Mother: htz | AR | Steenweg (2012)/ Steenweg (2012) |
| P27 | 3m ^†^ | M | RC:N | Metabolic acidosis with hyperlactacidemia; delayed psychomotor development; failure to thrive; increased transaminases; failure to thrive; high lactate and pyruvate; brain spectroscopy with lactate peak. | *EARS2* | p.Ile62Phe/ p.Leu307Ser | c.184A>T/ c.920T>C | D D | P D | D D | 24.0 23.9 | 3.18e^-5^ 1.25e^-5^ | 6.48e^-5^ (EUR) 5.93e^-5^ (LAT) | NA | AR | Oliveira (2017)/ Barbosa-Gouveia (2021) |
| P28 | 4y | F | NA | Hypotonia; ataxia; high lactate; MRI with bilateral basal ganglia involvement. | *EARS2* | p.Arg412His/ p.Lys471Asnfs*13 | c.1235G>A/ c.1413delG | T - | B - | D - | 22.8 - | 8.02e^-6^ 4.01e^-6^ | 8.84e^-6^ (EUR) 8,83e^-6^ (EUR) | Father: htz Mother: htz | AR | This study Güngör (2016) |
| P29 | 1m † | F | NA | Hyperlactacidemia; hiperaminoaciduria; congenital cardiomyopathy; neonatal diabetes; omphalocele. | *NARS2* | p.His167Arg/ p.Ala231_Ile320del | c.500A>G/ c.690-?_959+?del | D - | D - | D - | 25.4 - | 8.12e^-6^ - | 6.04e^-5^ (LAT) - | NA | AR | Mizuguchi (2017)/ This study |
| P30 | 1y † | F | NA | Generalized hypotonia; seizures; apneic episodes; cerebellar hypoplasia; microcephaly; vertical nystagmus. | *RARS2* | splicing/ splicing | c.451+4A>G/ c.451+4A>G | -  - | -  - | -  - | -  - | -  - | -  - | NA | AR | This study/ This study |
| P31 | 1y † | M | NA | Axial hypotonia; bradycardia; cerebellar hypoplasia; refractory epilepsy; feeding problems. | *RARS2* | splicing/ splicing | c.451+4A>G/ c.451+4A>G | -  - | -  - | -  - | -  - | -  - | -  - | NA | AR | This study/ This study |
| P32 | 1m † | F | NA | Fetal growth restriction and preterm birth; hypertrophic cardiomyopathy; hypotonia; metabolic acidosis with hyperlactacidemia. | *VARS2* | p.Ala360Val/ p.Ala420Thr | c.1079C>T/ c.1258G>A | T D | B D | N D | 22.9 29.3 | - 3.81e^-4^ | -  2.36e^-3^ (LAT) | NA | AR | This study/ Bruni (2018) |
| P33 | 23y | M | NA | Ophthalmoparesis; epilepsy; dystonia; tremor; MRI with basal ganglia calcifications. | *VARS2* | p.Thr367Ile/ p.Ala420Thr | c.1100C>T/ c.1258G>A | D D | D D | D D | 31 29.3 | 2.43e^-5^  3.81e^-4^ | 1.16^e-4^ (LAT) 2.36e^-3^ (LAT) | Mother: htz Father: htz | AR | Diodato (2014)/  Bruni (2018) |
| P34 | 15y | M | NA | Delayed psychomotor development; epilepsy; ataxia; brain MRI suspected of Leigh Syndrome; hyperlactacidemia. | *VARS2* | p.Ala594Val/ p.Thr647Met | c.1781C>T/ c.1940C>T | D D | B D | D D | 33 34 | 5.57e^-4^ 1.87e^-4^ | 2.59e^-3^ (EA) 2.27e^-3^ (EA) | NA | AR | This study/ Chin (2019) |
| P35 | 55y | F | NA | Dystonia; development delay; cerebral calcifications. | *WARS2* | p.Trp13Gly / p.Leu163Phe | c.37T>G/ c.487C>T | T D | B D | N D | 17.28 28.7 | 3.26e^-3^ 1.59e^-5^ | 4.58e^-3^ (EUR) 8.67e^-5^ (LAT) | Father: htz Mother: htz | AR | Burke (2018)/ Nogueira (2019) |
| P36 | 57y | M | NA | Bilateral optic atrophy. | *OPA1* | p.Pro400Ala | c.1198C>G | D | D | D | 28.4 | - | - | NA | AD | Zhang (2012) |
| P37 | 17y | M | NA | Bilateral optic neuropathy; family history of optic nerve atrophy. | *OPA1* | p.His631Argfs*2 | c.1892_1893delAT | - | - | - | - | - | - | NA | AD | Ferré (2009) |
| P38 | 67y | F | NA | Spastic paraparesis; cerebellar ataxia; chronic progressive external ophthalmoplegia. | *SPG7* | p.Gln483*/ p.Gln483* | c.1447C>T/ c.1447C>T/ | - - | - - | A A | 58 58 | 2.48e^-5^ 2.48e^-5^ | 2.4e^-4^ (AFR) 2.4e^-4^ (AFR) | NA | AR | Morais (2017)/ Morais (2017) |
| P39 | 4y | F | CI (40%) CII (23%) CIII (50%) CII+III (17%) CIV (31%) | Two episodes of cardiorrespiratory arrest during viral infections in the first 3 years of life; recurrent hyperlactacidemia; 3-methylgluctaconic aciduria, currently later assymptomatic. | *LYRM4* | p.Tyr31Cys/ p.Tyr31Cys | c.92A>G/ c.92A>G | D D | D D | D D | 26.9 26.9 | 3.18e^-5^ 3.18e^-5^ | 6.48e^-5^ (EUR) 6.48e^-5^ (EUR) | Father: htz Mother: htz | AR | Coelho (2019)/ Coelho (2019) |
| P40 | 3y | F | NA | Global developmental delay; dystonia; MRI with decreased signal intensity in the pallidal nuclei with focal T2 hyperintensity. | *PANK2* | splicing/ splicing | c.1537-3C>G/ c.1537-3C>G | - - | - - | - - | - - | - - | - - | NA | AR | Zhou (2001)/ Zhou (2001) |
| P41 | 2y | M | NA | Delayed psychomotor development; global hypotonia. | *PDHA1* | p.Phe205Leu | c.615C>G (hemi) | D | P | A | 24.8 | - | - | NA | XLD | Pavlu-Pereira (2020) |
| P42 | 19y | M | RC: N^a^ | Ataxia; cognitive impairment; epilepsy; high lactate, alanine and pyruvate. | *PDHX* | p.Arg284*/ p.Arg284* | c.850C>T/ c.850C>T | - - | - - | A A | 39 39 | 2.78e^-5^ 2.78e^-5^ | 1.39e^-4^ (EUR) 1.39e^-4^ (EUR) | Father: htz Mother: htz | AR | Pinheiro (2016)/ Pinheiro (2016) |
| P43 | 3y | F | RC: N^a^ | Ataxia; cognitive impairment; epilepsy. | *PDHX* | p.Arg284*/ p. Arg284* | c.850C>T/ c.850C>T | - - | - - | A A | 39 39 | 2.78e^-5^ 2.78e^-5^ | 1.39e^-4^ (EUR) 1.39e^-4^ (EUR) | Mother: htz Father: htz | AR | Pinheiro (2016)/ Pinheiro (2016) |
| P44 | 2y | F | NA | Epileptic encephalopathy responsive to biotin, riboflavin, thiamine and coQ. | *SLC19A3* | p.Ser26Leufs*18/ p.Ser26Leufs*18 | c.74dupT/  c.74dupT | - - | - - | - - | - - | 7.95e^-6^ 7.95e^-6^ | 1.76e^-5^ (EUR) 1.76e^-5^ (EUR) | NA | AR | Debs (2010)/ Debs (2010) |
| P45 | 6y | F | NA | Acute episodes of encephalitis with neurological deterioration; assymptomatic after thiamine and biotin suplementation. | *SLC19A3* | p.Trp59*/ splicing | c.177G>A/ c.980-14A>G | - - | - - | A - | 36 - | - 6.79e^-5^ | - 2.62e^-4^ (LAT) | Mother: htz Father: htz | AR | This study/ Debs (2010) |
| P46 | 4y | F | NA | Nystagmus; dystonia; global developmental delay; hypotonia; hyperlactacidemia. | *ECHS1* | p.Gly42Glufs*2/ p.Ala238Val | c.123_124del/ c.713C>T | - T | - D | - N | - 15.07 | 1.18e^-4^ 1.59e^-5^ | 1.98e^-4^ (SA) 3.51e^-5^ (EUR) | Mother: htz Father: htz | AR | Nogueira (2019)/ Tetreault (2015) |
| P47 | 7y | F | NA | Delayed psychomotor development; axial and peripheral hypotonia; cerebral and cerebellar atrophy; psychomotor regression. | *HIBCH* | p.Gly44Argfs*20/ p.Pro304Ser | c.129dupA/ c.910C>T | - D | - D | - D | - 29.4 | - - | - - | Mother: htz Father: htz | AR | Peters (2015)/ Baldo (2023) |
| P48 | 12y † | F | NA | Neurological involvement. ;delayed psychomotor evelopment; epilepsy; tetraparesis with some peripheral spasticity and axial hypotonia . | *CLN6* | p.Ala22Ser/ p.Ile154del | c.64G>T/ c.460_462delATC | T - | B - | N - | 16.82 - | 1.36e^-3^ 3.98e^-6^ | 1.33e^-2^ (AFR) 2.89e^-5^ (LAT) | Mother: htz Father: htz | AR | Nogueira (2019)/ Wheeler (2002) |
| P49 | 55y † | F | NA | Parkinsonism.; dyskinesias; rapidly progressive dementia | *KIF5A* | p.Arg716Gln | c.2147G>A | T | P | D | 28.0 | 2.48e^-5^ | 8.47e^-5^ (LAT) | NA | AD | Nakamura (2021) |
| P50 | 51y | M | NA | Basal ganglia calcification; white matter disease with cerebellar, pyramidal and dystonic syndromes. | *SPR* | p.Asp69Glu | c.207C>G | D | D | D | 28.3 | 1.21e^-4^ | 4.05e^-4^ (AFR) | NA | AD | Shalash (2017) |
| P51 | 14y | F | RC: N^a^ | Slowly progressive decreased visual acuity; optic atrophy; mild motor incoordination; MRI with cerebellum T2 hypersignal. | *TPP1* | p.Gln100Arg/ splicing | c.299A>G/ c.1266+5G>A | T - | B - | D - | 3.04 - | 2.86e^-3^ 6.16e^-3^ | 2.9e^-2^ (AFR) 6.18e^-2^ (AFR) | Mother: htz Father: htz | AR | Santorelli(2013)/ Nogueira (2019) |

^†^ Death; ^a^ citrate synthase increased; AD (Autosomal Dominant); AR (Autosomal Recessive); CADD (Combined Annotation Dependent Depletion); CI (Complex I); CII (Complex II); CIII (Complex III), CIV (Complex IV); CV (Complex V); CII+III (Complex II+III); CK (Creatine Kinase); Depl. (Depletion); Dx (Diagnosis); F (Female); gnomAD (Genome Aggregation Database, includes 123,136 exome and 15,496 whole genome sequences: “AFR” African; “AJ” Ashkenazi Jewish; “EA” East Asian; “EUR” European Non-Finnish; “LAT” Latino; “SA” South Asian); htz (heterozygous); m (months); M (Male); MRI (Magnetic Resonance Imaging); MT (Mutation Taster: “A” disease causing automatic, “D” disease causing, “N” polymorphism); N (Normal); NA (Not Available); PP2 (PolyPhen2 HDIV: “D” probably damaging, “P” possibly damaging, “B” benign); RC (Respiratory Chain); SIFT (“T” tolerated, “D” deleterious); y (years); Δ^M^ (multiple deletions of mtDNA).

All references cited in this table can be consulted in DataSheet 2.
