# Supplementary material for: The genetic landscape of mitochondrial diseases in the next-generation sequencing era: a Portuguese cohort study
Source: Front Cell Dev Biol. 2024 Feb 23;12:1331351. doi: 10.3389/fcell.2024.1331351 (PMC10920333; doi:10.3389/fcell.2024.1331351)
Supplement: Supplementary file 3 [file Table5.docx]

| Supplementary Table S5 – Variants of unknown significance (VUS) in mitochondrial DNA identified by next generation sequencing | | | | | | | | | | | | | | | |
| --- | --- | --- | --- | --- | --- | --- | --- | --- | --- | --- | --- | --- | --- | --- | --- |
|  | | | | | | | | | | | | | | | |
| **Patient** | **Dx**  **Age** | **Gender** | **Symptoms and signs** | **Gene** | **Nucleotide change** | **Protein**  **change** | **MTB** | **MC1** | **APG2** | **MTIP** | **Conservation** | **Mitomap Frequency**  **(61168 FL)** | **Homolasmy/ Heterolasmy**  **Segregation** | **References** |  |
| **P127** | 43y | F | Bilateral hypoacusis and hypovision. | *MT-RNR2* | m.2862C>T | - | - | - | - | - | 77.78% | - | (B) 45%  - | This study |  |
| **P128** | 17y | M | Neurological impairment | *MT-CO2* | m.7637G>A | p.Glu18Lys | D | D | VUS | - | 100.00% | 3.0e-5 | (B) 18%  - | Khusnutdinova (2008) |  |
| **P129** | 66y | M | Optic neuropathy. | *MT-ATP8* | m.8382C>T | p.Thr6Ile | D | D | LB | - | 84.44% | 1.5e^-4^ | (B) 36%  - | Rucheton (2020) |  |
| **P130** | 2y | M | Delayed psychomotor development; axial hypotonia. | *MT-ATP6* | m.8975T>C | p.Leu150Pro | D | D | VUS | - | 22.22% | 3.5e^-4^ | (B) 10%  (M) 10%  *De novo* | Rucheton (2020) |  |
| **P131** | 15y | M | Cardiomyopathy; tubulopathy; hyperlactacidemia. | *MT-CO3* | m.9331T>C | p.Leu42Pro | D | D | VUS | - | 100.00% | - | (B) Homo Mother:  (B) Homo | Nogueira (2019) |  |
| **P132** | 7y | F | Hyperinsulinism; delayed psychomotor development; epilepsy. | *MT-TG* | m.10011A>G | - | - | - | - | LP | 100.00% | - | (B) 10%  - | This study |  |
| **P133** | 1y | M | Hypoglycemia; seizures; thrombocytopenia. | *MT-ND5* | m.12425delA | p.Asn30Thrfs*7 | - | - | - | - | 28.89% | 7.0e^-5^ | (B) 12%  - | Alston (2010) |  |
| **P134** | 11y | F | Short stature; mild delayed psychomotor development; tubulopathy; retinitis pigmentosa; mild hypoacusis; organic acid profile alterations; hyperlactacidemia; panhypopituitarism; Ragged-Red-Fibers and COX deficiency. | *MT-ND5* | m.13115T>C | p.Leu260Pro | D | D | LP | - | 93.33% | - | (B): 82%  (BM) - -  (M) - - | This study |  |

APG 2 (APOGEE: “B” benign, “LB” likely benign, “VUS” variant of unknown significance, “LP” likely pathogenic, “P” pathogenic); (B) – Blood; COX (Cytochrome c Oxidase); (BM) – Buccal mucosa; Dx (Diagnosis); F (Female); FL (Full Length Sequences); m (months); M (Male); MC1 (MitoClass 1: “N” Neutral, “D” Damaging); (M) – Muscle; MRI (Magnetic resonance imaging); MTIP (MitoTIP: “LB” likely benign, “PB” possibly benign, “PP” possibly pathogenic, “LP” likely pathogenic, “CP” confirmed pathogenic); w (weeks); y (years).

All references cited in this table can be consulted in DataSheet 2.
