# Supplementary material for: The genetic landscape of mitochondrial diseases in the next-generation sequencing era: a Portuguese cohort study
Source: Front Cell Dev Biol. 2024 Feb 23;12:1331351. doi: 10.3389/fcell.2024.1331351 (PMC10920333; doi:10.3389/fcell.2024.1331351)
Supplement: Supplementary file 4 [file Table3.docx]

| Supplementary Table S3 – Variants of unknown significance (VUS) identified in nuclear DNA by next generation sequencing. | | | | | | | | | | | | | | | | |
| --- | --- | --- | --- | --- | --- | --- | --- | --- | --- | --- | --- | --- | --- | --- | --- | --- |
|  | | | | | | | | | | | | | | | | |
| Patient | **Dx Age** | **Gender** | **Muscular Defects** | **Clinical Presentation** | **Gene** | **Protein change** | **Nucleotide change** | **SIFT** | **PP2** | **MT** | **CADD** | **gnomAD (Overall)** | **gnomAD (Highest freq)** | **Segregation Data** | **Trait** | **References** |
| P52 | 3y | F | NA | Bilateral frontoparietal polymicrogyria; seizures; axial hypotonia. | *ATP5F1A* | p.Ser236Cys/ p.Val417Val | c.707C>G/ c.1251C>T | D - | B - | D - | 23.6 - | - 2.12e^-5^ | - 8.01e^-5^ (AFR) | NA | AR | This study/ This study |
| P53 | 4y | F | NA | Mitochondrial cytopathy. | *NDUFS6* | p.Glu62Glu/ p.Glu62Glu | c.186G>A/ c.186G>A | - - | - - | - - | - - | - - | - - | Father: htz Mother: htz | AR | This study/ This study |
| P54 | 42Y | M | NA | MRI suspected of Leigh syndrome; short stature. | *NDUFS8* | p.Arg66*/ p.Arg96His | c.196C>T/ c.287G>A | - D | - D | A D | 37 26.6 | - - | - - | Father:htz Mother:htz | AR | Nogueira (2019)/ Nogueira (2019) |
| P55 | 51y | F | NA | Deafness; MRI brain alterations; leukoencephalopathy. | *ACAD9* | p.Lys51Ile/ p.Leu362Phe | c.152A>T/ c.1084C>T | T D | P B | D D | 22.4 32 | 2.17e^-4^  3.98e^-6^ | 1.26e^-3^ (AJ) 2.89e^-5^ (LAT) | NA | AR | Nogueira (2019)/ Nogueira (2019) |
| P56 | 6y | M | RC: N | Myopathy. | *FASTKD2* | p.Ser119Phe/ p.Leu399Phe | c.356C>T/ c.1195C>T | T D | B P | N D | 7.96 24.9 | 9.21e^-5^ 3.1e^-4^ | 2.54e^-4^ (LAT) 1.59e^-3^ (LAT) | Father: htz Mother: htz | AR | Nogueira (2019)/ Nogueira (2019) |
| P57 | 16y | F | CIV (48%)^a^ | Delayed psychomotor; development; dysmorphism; axonal neuropathy; early puberty. | *TMEM70* | p.Asn228Lys/ p.Asn228Lys | c.684C>G/ c.684C>G | D D | P P | D D | 8.23 8.23 | 2.06e^-3^ 2.06e^-3^ | 1.73e^-2^ (AFR) 1.73e^-2^ (AFR) | Mother: htz | AR | Nogueira (2019)/ Nogueira (2019) |
| P58 | 31y | F | NA | Cardiomyopathy. | *GTPBP3* | p.Glu157Val/ p.Glu157Val | c.470A>T/ c.470A>T | D D | D D | D D | 29.9 29.9 | - - | - - | NA | AR | This study/ This study |
| P59 | 11y | F | CII, CIV, CV; CII+III | Cognitive impairment; lower limbs atrophy; MCAD deficiency | *GTPBP3* | p.Arg368His/ p.Arg368His | c.1103G>A/ c.1103G>A | T T | B B | N N | 22.7 22.7 | 6.94e^-2^ 6.94e^-2^ | 3.22e^-1^ (EA) 3.22e^-1^ (EA) | NA | AR | Nogueira (2019)/ Nogueira (2019) |
| P60 | 5y | M | CI (27%), CIV (42%)^a^ | Neurological and muscular involvement; deafness; short stature; hypotonia. | *POLG* | splicing | c.1170+5G>C | - | - | - | - | - | - | NA | AD | Nogueira (2019) |
| P61 | 9y | M | CIV, CV | Failure to thrive; delayed psychomotor development; feeding difficulties; hypotonia. | *POLG* | p.Thr858Ile | c.2573C>T | D | D | D | 33 | 2.39e^-5^ | 5.28e^-5^ (EUR) | Mother: htz Father: N | AD | Nogueira (2019) |
| P62 | 1y | F | NA | Irritability; feeding difficulties; involuntary movements of the lower limbs; epilepsy. | *POLG2* | p.Ser178Tyr | c.533C>A | T | D | D | 24.6 | - | - | NA | AD | This study |
| P63 | 57y | F | Δ^M^ | Cerebellar ataxia; spastic paraparesis since age 33; polyneuropathy; sensorineural deafness. | *POLG2* | splicing | c.1293-3delT | - | - | - | - | - | - | NA | AD | This study |
| P64 | 17y | M | NA | Delayed psychomotor development; speech delay; obesity. | *CARS2* | splicing/ p.Val497Ile/ p.Lys561Glu | c.572-3_572-2delCA / c.1489G>A/ c.1681A>G | - T D | - P B | - D N | - 24.8 13.3 | 3.03e^-3^ 1.07e^-3^ 1.69e^-3^ | 3.07e^-2^ (AFR) 1.14e^-2^ (AFR)  1.77e^-2^ (AFR) | NA | AR | This study/ This study/ This study |
| P65 | 2y | M | NA | Hyperlactacidemia; hyperalaninemia; hypotonia; mitochondrial dysfunction; non progresive hypotonia, predomoninantly in lower limbs; high lactate and alanine; normal MRI. | *DARS2/ KARS1* | p.Tyr30*/ p.Arg537Trp | c.90C>A/ c.1609C>T | - D | - D | A D | 32 31 | - 1.06e^-5^ | -  2.32e^-5^ (EUR) | Mother: htz Father: htz | AR/ AR | Stellingwerff (2021) / This study |
| P66 | 14y | F | RC: N;  Depl (60%) | Neurological and muscular involvement; aggressive behavior | *LARS2* | p.Asp649Glu/ p.Gln858Lys | c.1947C>A/ c.2572C>A | T T | B B | D N | 16.28 16.20 | 5.3e^-5^ 2.82e^-5^ | 4.4e^-4^ (AFR) 5.54e^-5^ (EA) | NA | AR | Nogueira (2019)/ Nogueira (2019) |
| P67 | 7y ^†^ | F | NA | Encephalopathy; renal insufficiency; elevation of transaminases; hepatomegaly; arterial hypertension; ventricular septal defect; increased CK; rhabdomyolysis. | *LARS2* | p.Glu831Asp/ p.Glu868Lys | c.2493G>T/ c.2602G>A | T T | B D | D D | 9.79 18.88 | 3.86e^-3^ 8.78e^-4^ | 3.98e^-2^ (AFR) 8.26e^-3^ (AFR) | NA | AR | This study/ This study |
| P68 | 11y | M | RC: N; RRFs | Delayed psychomotor development; epilepsy; preterm; normal MRI, without tubulopathy. | *SARS2* | p.Ser83Leu/ p.Ser83Leu | c.248C>T/ c.248C>T | T T | P P | - - | 9.37 9.37 | 9.04e^-3^ 9.04e^-3^ | 2.62e^-2^ (SA) 2.62e^-2^ (SA) | NA | AR | Nogueira (2019)/ Nogueira (2019) |
| P69 | 5y | M | NA | Axial hypotonia with peripheral hypertonia; delayed psychomotor development. | *VARS2* | p.Gly233Arg/ p.Gly233Arg | c.697G>A/ c.697G>A | T T | B B | D D | 21.8 21.8 | 1.26e^-3^ 1.26e^-3^ | 1.22e^-2^ (AFR) 1.22e^-2^ (AFR) | Mother: htz | AR | This study/  This study |
| P70 | 78y | F | RC: N | PEO; vocal fold paresis. | *MTO1* | splicing/ splicing | c.1918-7T>G/ c.1918-7T>G | -  - | -  - | -  - | - - | 1.4e^-2^ 1.4e^-2^ | 1.4e^-1^ (AFR) 1.4e^-1^ (AFR) | NA | AR | Nogueira (2019)/ Nogueira (2019) |
| P71 | 3y | M | NA | Delayed psychomotor development; hyperlactacidemia. | *TSFM* | p.Arg8Arg/ p.Arg275Cys | c.24C>T/ c.823C>T | - D | - D | - D | - 34 | 1.5e^-3^ 8.54e^-4^ | 1.37e^-2^ (AJ) 1.37e^-2^ (AJ) | NA | AR | This study/ This study |
| P72 | 2y | M | NA | Mitochondrial cytopathy. | *TSFM/ GFM1* | p.Arg275Cys/ p.Val125Met | c.823C>T/ c.373G>A | D D | D D | D D | 34 29.1 | 8.54e^-4^ 2.77e^-4^ | 1.37e^-2^ (AJ) 6.37e^-3^ (AJ) | NA | AR/ AR | This study/ This study |
| P73 | 14y | M | NA | Degenerative disease of the central nervous system; gait alteration; speech and fine motor disorders; difficulties in eating; cognitive decline. | *MFN2* | p.Thr423Ala | c.1267A>G | T | B | D | 21.8 | 1.19e^-5^ | 8.67e^-5^ (LAT) | Father: htz | AD | This study |
| P74 | 19y | F | NA | Imbalance in gait and incoordination in motor activities; slight ataxia; visual difficulty; cognitive alteration. | *MFN2* | p.Thr423Ala | c.1267A>G | T | B | D | 21.8 | 1.19e^-5^ | 8.67e^-5^ (LAT) | Father: htz | AD | This study |
| P75 | 12h^†^ | F | Depl (80%) | Anemia; acidosis; pulmonary hypertension; neonatal seizures; neonatal thrombocytopenia; hepatomegaly, hypertrophic cardiomiopathy. | *SLC25A3* | p.Thr16Met | c.47C>T | T | B | D | 22.6 | 4.97e^-5^ | 5.23e^-4^ (AFR) | Mother: htz | AD | Nogueira (2019) |
| P76 | 8y | M | RC: N^a^ | Congenital ataxia; hypotonia; microcephaly. | *TAFAZZIN* | p.Thr18Ile | c.53C>T | T | D | N | 20.08 | - | - | NA | XLR | Nogueira (2019) |
| P77 | 15y | M | NA | Dilated cardiomyopathy. | *COQ8A* | p.Thr519Ser/ p.Ala571Thr | c.1556C>G/ c.1711G>A | D T | P P | D D | 27.5 23.2 | 4.95e^-5^  2.23e^-4^ | 1.13e^-4^ (LAT) 2.91e^-2^ (EA) | NA | AR | This study/ This study |
| P78 | 2Y | F | NA | Neurological involvement; hypotonia; delayed psychomotor development; seizures; failure to thrive. | *DLD* | Splicing/ splicing | c.40-31C>T/ c.40-31C>T | - - | - - | - - | - - | 1.21e^-5^ 1.21e^-5^ | 2.67e^-5^ (EUR) 2.67e^-5^ (EUR) | Father: htz Mother: htz | AR | This study/  This study |
| P79 | 18y | F | CII+ CIII  (23%) | Liver dysfunction; reye-Like syndrome; delayed psychomotor development; epilepsy; extrapyramidal syndrome; MRI brain alterations; hyperlactacidemia. | *DLD* | p.Pro87Ser/ p.Gln268Argfs*3 | c.259C>T/ c.803_804delAG | D - | D - | D - | 31 - | - 1.19e^-5^ | - 3.27e^-5^ (SA) | NA | AR | Bravo-Alonso(2019)/ Pavlu-Pereira(2020) |
| P80 | 12y | M | NA | Non compaction cardiomyopathy and long QT syndrome; father with same issues; normal transferrin isoelectric focusing. | *ALG13* | splicing | c.933-4A>G (hemi) | - | - | - | - | 1.7e^-5^ | 5.89e^-5^ (AFR) | Mother: htz | XLD | This study |
| P81 | 7y | F | NA | Intellectual disability; behavior slterations; microcephaly; hypotonia; facial dysmorphia. | *CWF19L1* | splicing p.Gly86Glu | c.109-46_109-45delTT/ c.257G>A | - D | - D | - D | - 32 | 7.0^-4^ 6.42e^-4^ | 7.24e^-3^ (AFR) 6.82e^-3^ (AFR) | NA | AR | This study/ This study |
| P82 | 10y | M | NA | Neurological involvement. | *DNAJC5* | p.Ala54Val | c.161C>T | D | P | D | 25.8 | 7.96e^-6^ | 6.16e^-5^ (AFR) | NA | AD | This study |
| P83 | 10y | M | NA | Neurological involvement; delayed psychomotor development. | *KIF5A* | p.Met96Leu | c.286A>T | D | B | D | 27.8 | - | - | NA | AD | This study |
| P84 | 3y | M | NA | Growth retardation; feeding difficulties; neonatal hypoglycemia | *KIF5A* | p.Lys744Asn | c.2232G>T | D | P | D | 22.7 | 3.98e^-6^ | 2.89e^-5^ (LAT) | NA | AD | Nogueira (2019) |
| P85 | 13y | F | NA | Epilepsy; learning difficulties; hypoplasia of the vermis and cerebellar hemispheres. | *MFSD8* | splicing/ p.Pro356Thr | c.863+4A>G/ c.1066C>A | - D | - D | - D | - 27.4 | 1.77e^-5^ 1.59e^-5^ | 3.88e^-5^ (EUR) 2.89e^-5^ (EUR) | NA | AR | Jilani (2019)/ Dozieres-Puyravel (2020) |
| P86 | 51y | F | NA | Epilepsy; delay; extrapyramidal dystonia; deafness. | *NOTCH3* | p.His555Arg | c.1664A>G | T | B | D | 22.9 | - | - | NA | AD | This study |
| P87 | 7m | M | NA | Neurological involvement; axial hypotonia; psychomotor development retardation; seizures; strabismus; | *TPP1* | splicing/ p.Pro188Leu | c.509-6C>G/ c.563C>T | - T | - B | - D | - 15.07 | 7.96e^-6^ 4.25e^-5^ | 1.09e^-4^ (EA) 2.79e^-4^ (FIN) | NA | AR | Nogueira (2019)/ Nogueira (2019) |
| P88 | 26y | F | NA | Diabetes; optic atrophy; deafness. | *WFS1* | p.Ala569Val/ p.Ala602Val | c.1706C>T/ c.1805C>T | T T | P B | D N | 22.1 0.001 | 1.63e^-4^ 9.3e^-3^ | 1.69e^-3^ (AFR) 9.15e^-2^ (AFR) | NA | AR | Chaussenot (2015)/ Torres (2001) |

^†^ Death; ^a^ citrate synthase increased; AD (Autosomal Dominant); AR (Autosomal Recessive); CADD (Combined Annotation Dependent Depletion); CI (Complex I); CII (Complex II); CIV (Complex IV); CV (Complex V); CII+III (Complex II+III); CK (Creatine Kinase); Depl (mtDNA Depletion); Dx (Diagnosis); F (Female); gnomAD (Genome Aggregation Database, includes 123,136 exome and 15,496 whole genome sequences: “AFR” African; “AJ” Ashkenazi Jewish; “EA” East Asian; “EUR” European Non-Finnish; “FIN” European Finnish; “LAT” Latino; “SA” South Asian); h (hours); htz (heterozygous); m (months); M (Male); MCAD (Medium-Chain Acyl-CoA Dehydrogenase); MRI (Magnetic Resonance Imaging); mt (mitochondrial inheritance); MT (Mutation Taster: “A” disease causing automatic, “D” disease causing, “N” polymorphism, “P” polymorphism automatic); N (Normal); NA (Not Available); PEO (Progressive External Ophthalmoplegia); PP2 (PolyPhen2 HDIV: “D” probably damaging, “P” possibly damaging, “B” benign); RC (Respiratory Chain); RRFs (Ragged-Red Fibers); SIFT (“T” tolerated, “D” deleterious); XLR (X-Linked Recessive); y (years); Δ^M^ (multiple deletions of mtDNA).

All references cited in this table can be consulted in DataSheet 2.
