# Supplementary material for: The genetic landscape of mitochondrial diseases in the next-generation sequencing era: a Portuguese cohort study
Source: Front Cell Dev Biol. 2024 Feb 23;12:1331351. doi: 10.3389/fcell.2024.1331351 (PMC10920333; doi:10.3389/fcell.2024.1331351)
Supplement: Supplementary file 5 [file DataSheet2.docx]

**Supplementary Table S2 References**

Baldo, M. S., Nogueira, C., Pereira, C., Janeiro, P., Ferreira, S., Lourenço, C. M., et al. (2023). Leigh Syndrome Spectrum: A Portuguese Population Cohort in an Evolutionary Genetic Era. *Genes*, 14(8), 1536. doi:10.3390/genes14081536

Barbosa-Gouveia, S., Vázquez-Mosquera, M. E., González-Vioque, E., Álvarez, J. V., Chans, R., Laranjeira, F., et al. (2021). Utility of Gene Panels for the Diagnosis of Inborn Errors of Metabolism in a Metabolic Reference Center. *Genes*, 12(8), 1262. doi:10.3390/genes12081262

Bénit, P., Beugnot, R., Chretien, D., Giurgea, I., De Lonlay-Debeney, P., Issartel, J.P., et al. (2003). Mutant NDUFV2 subunit of mitochondrial complex I causes early onset hypertrophic cardiomyopathy and encephalopathy. *Hum. Mutat.* 21(6), 582-586. doi:10.1002/humu.10225

Bruni, F., Di Meo, I., Bellacchio, E., Webb, B. D., McFarland, R., Chrzanowska-Lightowlers, Z. M. A., et al. (2018). Clinical, biochemical, and genetic features associated with VARS2-related mitochondrial disease. *Hum. Mutat*., 39(4), 563–578. doi:10.1002/humu.23398

Burke, E. A., Frucht, S. J., Thompson, K., Wolfe, L. A., Yokoyama, T., Bertoni, M., et al. (2018). Biallelic mutations in mitochondrial tryptophanyl-tRNA synthetase cause Levodopa-responsive infantile-onset Parkinsonism. *Clin. Genet.*, 93(3), 712–718. doi:10.1111/cge.13172

Chin, J., Marotta, R., Chiotis, M., Allan, E. H., and Collins, S. J. (2014). Detection rates and phenotypic spectrum of m.3243A>G in the MT-TL1 gene: a molecular diagnostic laboratory perspective. *Mitochondrion*, 17, 34–41. doi:10.1016/j.mito.2014.05.005

Coelho, M. P., Correia, J., Dias, A., Nogueira, C., Bandeira, A., Martins, E., and Vilarinho, L. (2019). Iron-sulfur cluster ISD11 deficiency (LYRM4 gene) presenting as cardiorespiratory arrest and 3-methylglutaconic aciduria. *JIMD reports*, 49(1), 11–16. doi:10.1002/jmd2.12058

Debs, R., Depienne, C., Rastetter, A., Bellanger, A., Degos, B., Galanaud, D., et al. (2010). Biotin-responsive basal ganglia disease in ethnic Europeans with novel SLC19A3 mutations. *Arch. Neurol.,* 67(1), 126–130. doi:10.1001/archneurol.2009.293

Diodato, D., Melchionda, L., Haack, T. B., Dallabona, C., Baruffini, E., Donnini, C., et al. (2014). VARS2 and TARS2 mutations in patients with mitochondrial encephalomyopathies. *Hum. Mutat*., 35(8), 983–989. doi:10.1002/humu.22590

Emperador, S., Bayona-Bafaluy, M. P., Fernández-Marmiesse, A., Pineda, M., Felgueroso, B., López-Gallardo, E., et al. (2016). Molecular-genetic characterization and rescue of a TSFM mutation causing childhood-onset ataxia and nonobstructive cardiomyopathy. *Eur. J. Hum. Genet*., 25(1), 153–156. doi:10.1038/ejhg.2016.124

Fernandez-Vizarra, E., Bugiani, M., Goffrini, P., Carrara, F., Farina, L., Procopio, E., et al. (2007). Impaired complex III assembly associated with BCS1L gene mutations in isolated mitochondrial encephalopathy. *Hum*. *Mol*. *Genet*., 16(10), 1241–1252. doi:10.1093/hmg/ddm072

Ferré, M., Bonneau, D., Milea, D., Chevrollier, A., Verny, C., Dollfus, H., et al. (2009). Molecular screening of 980 cases of suspected hereditary optic neuropathy with a report on 77 novel OPA1 mutations. *Hum. Mutat.*, 30(7), E692–E705. doi:10.1002/humu.21025

Garone, C., Taylor, R. W., Nascimento, A., Poulton, J., Fratter, C., Domínguez-González, C., et al. (2018). Retrospective natural history of thymidine kinase 2 deficiency. *J. Med. Genet.*, 55(8), 515–521. doi:10.1136/jmedgenet-2017-105012

Gil-Borlado, M. C., González-Hoyuela, M., Blázquez, A., García-Silva, M. T., Gabaldón, T., Manzanares, J., et al. (2009). Pathogenic mutations in the 5' untranslated region of BCS1L mRNA in mitochondrial complex III deficiency. *Mitochondrion*, 9(5), 299–305. doi:10.1016/j.mito.2009.04.001

Güngör, O., Özkaya, A. K., Şahin, Y., Güngör, G., Dilber, C., and Aydın, K. (2016). A compound heterozygous EARS2 mutation associated with mild leukoencephalopathy with thalamus and brainstem involvement and high lactate (LTBL). *Brain Dev.*, 38(9), 857–861. doi:10.1016/j.braindev.2016.04.002

Hinson, J. T., Fantin, V. R., Schönberger, J., Breivik, N., Siem, G., McDonough, B., et al. (2007). Missense mutations in the BCS1L gene as a cause of the Björnstad syndrome. *N. Engl. J. Med.*, 356(8), 809–819. doi:10.1056/NEJMoa055262

Huang, X., Bedoyan, J. K., Demirbas, D., Harris, D. J., Miron, A., Edelheit, S., et al. (2017). Succinyl-CoA synthetase (SUCLA2) deficiency in two siblings with impaired activity of other mitochondrial oxidative enzymes in skeletal muscle without mitochondrial DNA depletion. *Mol. Genet. Metab.,* 120(3), 213–222. doi:10.1016/j.ymgme.2016.11.005

Huemer, M., Karall, D., Schossig, A., Abdenur, J. E., Al Jasmi, F., Biagosch, C., et al. (2015). Clinical, morphological, biochemical, imaging and outcome parameters in 21 individuals with mitochondrial maintenance defect related to FBXL4 mutations. *J. Inherit. Metab. Dis.*, 38(5), 905–914. doi.org/10.1007/s10545-015-9836-6

Invernizzi, F., Tigano, M., Dallabona, C., Donnini, C., Ferrero, I., Cremonte, M., et al. (2013). A homozygous mutation in LYRM7/MZM1L associated with early onset encephalopathy, lactic acidosis, and severe reduction of mitochondrial complex III activity. *Hum*. *Mutat*, 34(12), 1619–1622. doi:10.1002/humu.22441

Lamantea, E., Tiranti, V., Bordoni, A., Toscano, A., Bono, F., Servidei, S., et al. (2002). Mutations of mitochondrial DNA polymerase gammaA are a frequent cause of autosomal dominant or recessive progressive external ophthalmoplegia. *Ann. Neurol*., 52(2), 211–219. doi:10.1002/ana.10278

Mizuguchi, T., Nakashima, M., Kato, M., Yamada, K., Okanishi, T., Ekhilevitch, N., et al. (2017). PARS2 and NARS2 mutations in infantile-onset neurodegenerative disorder. *J. Hum. Genet.*, 62(5), 525–529. doi:10.1038/jhg.2016.163

Morais, S., Raymond, L., Mairey, M., Coutinho, P., Brandão, E., Ribeiro, P., et al. (2017). Massive sequencing of 70 genes reveals a myriad of missing genes or mechanisms to be uncovered in hereditary spastic paraplegias. *Eur. J. Hum. Genet.*, 25(11), 1217–1228. doi:10.1038/ejhg.2017.124

Nakamura, R., Tohnai, G., Atsuta, N., Nakatochi, M., Hayashi, N., Watanabe, H., et al. (2021). Genetic and functional analysis of KIF5A variants in Japanese patients with sporadic amyotrophic lateral sclerosis. *Neurobiol. Aging*, 97, 147.e11–147.e17. doi:10.1016/j.neurobiolaging.2020.07.010

Nogueira, C., Silva, L., Pereira, C., Vieira, L., Leão Teles, E., Rodrigues, E., et al. (2019). Targeted next generation sequencing identifies novel pathogenic variants and provides molecular diagnoses in a cohort of pediatric and adult patients with unexplained mitochondrial dysfunction. *Mitochondrion*, 47:309-317. doi: 10.1016/j.mito.2019.02.006

Nouws, J., Nijtmans, L., Houten, S.M., van den Brand, M., Huynen, M., Venselaar, H., et al. (2010). Acyl-CoA dehydrogenase 9 is required for the biogenesis of oxidative phosphorylation complex I. *Cell Metab.* 12(3), 283-294. doi:10.1016/j.cmet.2010.08.002

O'Byrne, J. J., Tarailo-Graovac, M., Ghani, A., Champion, M., Deshpande, C., Dursun, A., et al. (2018). The genotypic and phenotypic spectrum of MTO1 deficiency. *Mol. Genet. Metab.*, 123(1), 28–42. doi:10.1016/j.ymgme.2017.11.003

Oliveira, R., Sommerville, E. W., Thompson, K., Nunes, J., Pyle, A., Grazina, M., et al. (2017). Lethal Neonatal LTBL Associated with Biallelic EARS2 Variants: Case Report and Review of the Reported Neuroradiological Features. *JIMD reports*, 33, 61–68. doi:10.1007/8904_2016_581

Pavlu-Pereira, H., Silva, M. J., Florindo, C., Sequeira, S., Ferreira, A. C., Duarte, S., et al. (2020). Pyruvate dehydrogenase complex deficiency: updating the clinical, metabolic and mutational landscapes in a cohort of Portuguese patients. *Orphanet J. Rare Dis.*, 15(1), 298. doi:10.1186/s13023-020-01586-3

Peters, H., Ferdinandusse, S., Ruiter, J. P., Wanders, R. J., Boneh, A., and Pitt, J. (2015). Metabolite studies in HIBCH and ECHS1 defects: Implications for screening. *Mol. Genet. Metab.,* 115(4), 168–173. doi:10.1016/j.ymgme.2015.06.008

Pinheiro, A., Silva, M. J., Pavlu-Pereira, H., Florindo, C., Barroso, M., Marques, B., et al. (2016). Complex genetic findings in a female patient with pyruvate dehydrogenase complex deficiency: Null mutations in the PDHX gene associated with unusual expression of the testis-specific PDHA2 gene in her somatic cells. *Gene*, 591(2), 417–424. doi:10.1016/j.gene.2016.06.041

Santorelli, F.M., Garavaglia, B., Cardona, F., Nardocci, N., Bernardina, D., Sartori S., et al. (2013). Molecular epidemiology of childhood neuronal ceroid-lipofuscinosis in Italy. Orphanet J Rare Dis, 8:19. doi: 10.1186/1750-1172-8-19.

Scheper, G. C., van der Klok, T., van Andel, R. J., van Berkel, C. G., Sissler, M., Smet, J., et al. (2007). Mitochondrial aspartyl-tRNA synthetase deficiency causes leukoencephalopathy with brain stem and spinal cord involvement and lactate elevation. *Nat. Genet.*, 39(4), 534–539. doi:10.1038/ng2013

Shalash, A. S., Rösler, T. W., Müller, S. H., Salama, M., Deuschl, G., Müller, U., et al. (2017). c.207C>G mutation in sepiapterin reductase causes autosomal dominant dopa-responsive dystonia. *Neurol*. *Genet*., 3(6), e197. doi:10.1212/NXG.0000000000000197

Slavotinek, A. M., Garcia, S. T., Chandratillake, G., Bardakjian, T., Ullah, E., Wu, D., et al. (2015). Exome sequencing in 32 patients with anophthalmia/microphthalmia and developmental eye defects. *Clin. Genet.*, 88(5), 468–473. doi:10.1111/cge.12543

Steenweg, M. E., Ghezzi, D., Haack, T., Abbink, T. E., Martinelli, D., van Berkel, C. G., et al. (2012). Leukoencephalopathy with thalamus and brainstem involvement and high lactate 'LTBL' caused by EARS2 mutations. *Brain*, 135(5), 1387–1394. doi:10.1093/brain/aws070

Tiranti, V., Hoertnagel, K., Carrozzo, R., Galimberti, C., Munaro, M., Granatiero, M., et al. (1998). Mutations of SURF-1 in Leigh disease associated with cytochrome c oxidase deficiency. *Am. J. Hum. Genet.*, 63(6), 1609–1621. doi:10.1086/302150

Tetreault, M., Fahiminiya, S., Antonicka, H., Mitchell, G. A., Geraghty, M. T., Lines, M., et al. (2015). Whole-exome sequencing identifies novel ECHS1 mutations in Leigh syndrome. *Hum. Genet.*, 134(9), 981–991. doi:10.1007/s00439-015-1577-y

Van Goethem, G., Schwartz, M., Löfgren, A., Dermaut, B., Van Broeckhoven, C., and Vissing, J. (2003). Novel POLG mutations in progressive external ophthalmoplegia mimicking mitochondrial neurogastrointestinal encephalomyopathy*. Eur. J. Hum. Genet*., 11(7), 547–549. doi:10.1038/sj.ejhg.5201002

Wheeler, R. B., Sharp, J. D., Schultz, R. A., Joslin, J. M., Williams, R. E., and Mole, S. E. (2002). The gene mutated in variant late-infantile neuronal ceroid lipofuscinosis (CLN6) and in nclf mutant mice encodes a novel predicted transmembrane protein. *Am. J. Hum. Genet.*, 70(2), 537–542. https://doi.org/10.1086/338708

Zhang, J., Yuan, Y., Lin, B., Feng, H., Li, Y., Dai, X., et al. (2012). A novel OPA1 mutation in a Chinese family with autosomal dominant optic atrophy. *Biochem. Biophys. Res. Commun.*, 419(4), 670–675. doi:10.1016/j.bbrc.2012.02.073

Zhou, B., Westaway, S. K., Levinson, B., Johnson, M. A., Gitschier, J., and Hayflick, S. J. (2001). A novel pantothenate kinase gene (PANK2) is defective in Hallervorden-Spatz syndrome. *Nat. Genet.*, 28(4), 345–349. doi:10.1038/ng572

**Supplementary Table S3 References**

Bravo-Alonso, I., Navarrete, R., Vega, A. I., Ruíz-Sala, P., García Silva, M. T., Martín-Hernández, E., et al. (2019). Genes and variants underlying human congenital lactic acidosis – from genetics to personalized treatment. *J. Clin. Med.*, 8(11), 1811. doi:10.3390/jcm8111811

Chaussenot, A., Rouzier, C., Quere, M., Plutino, M., Ait-El-Mkadem, S., Bannwarth, et al. (2015). Mutation update and uncommon phenotypes in a French cohort of 96 patients with WFS1-related disorders. *Clin. Genet.*, 87(5), 430–439. doi:10.1111/cge.12437

Dozières-Puyravel, B., Nasser, H., Elmaleh-Bergès, M., Lopez Hernandez, E., Gelot, A., Ilea, A., et al., (2020). Paediatric-onset neuronal ceroid lipofuscinosis: first symptoms and presentation at diagnosis. *Dev Med Child Neurol*, 62(4):528-530. doi: 10.1111/dmcn.14346.

Jilani, A., Matviychuk, D., Blaser, S., Dyack, S., Mathieu, J., Prasad, A. N., et al. (2019). High diagnostic yield of direct Sanger sequencing in the diagnosis of neuronal ceroid lipofuscinoses. *JIMD reports*, 50(1), 20–30. doi:10.1002/jmd2.12057

Nogueira, C., Silva, L., Pereira, C., Vieira, L., Leão Teles, E., Rodrigues, E., et al. (2019). Targeted next generation sequencing identifies novel pathogenic variants and provides molecular diagnoses in a cohort of pediatric and adult patients with unexplained mitochondrial dysfunction. *Mitochondrion*, 47:309-317. doi: 10.1016/j.mito.2019.02.006

Pavlu-Pereira, H., Silva, M. J., Florindo, C., Sequeira, S., Ferreira, A. C., Duarte, S., et al. (2020). Pyruvate dehydrogenase complex deficiency: updating the clinical, metabolic and mutational landscapes in a cohort of Portuguese patients. *Orphanet J. Rare Dis.*, 15(1), 298. doi:10.1186/s13023-020-01586-3

Stellingwerff, M. D., Figuccia, S., Bellacchio, E., Alvarez, K., Castiglioni, C., Topaloglu, P., et al. (2021). LBSL: Case series and DARS2 variant analysis in early severe forms with unexpected presentations. *Neurol. Genet*., 7(2), e559. doi:10.1212/NXG.0000000000000559

Torres, R., Leroy, E., Hu, X., Katrivanou, A., Gourzis, P., Papachatzopoulou, A., et al. (2001). Mutation screening of the Wolfram syndrome gene in psychiatric patients. *Mol.* *Psychiatry*, 6(1), 39–43. doi:10.1038/sj.mp.4000787

**Supplementary Table S4 References**

Carrozzo, R., Murray, J., Santorelli, F. M., and Capaldi, R. A. (2000). The T9176G mutation of human mtDNA gives a fully assembled but inactive ATP synthase when modeled in Escherichia coli. *FEBS letters*, *486*(3), 297–299. doi:10.1016/s0014-5793(00)02244-4

Goto, Y., Nonaka, I., and Horai, S. (1990). A mutation in the tRNA(Leu)(UUR) gene associated with the MELAS subgroup of mitochondrial encephalomyopathies. *Nature*, *348*(6302), 651–653. doi:10.1038/348651a0

Goto, Y., Nonaka, I., and Horai, S. (1991). A new mtDNA mutation associated with mitochondrial myopathy, encephalopathy, lactic acidosis and stroke-like episodes (MELAS). *Biochim Biophys Acta*, 1097(3), 238–240. doi:10.1016/0925-4439(91)90042-8

Holt, I. J., Harding, A. E., Petty, R. K., and Morgan-Hughes, J. A. (1990). A new mitochondrial disease associated with mitochondrial DNA heteroplasmy. *Am. J. Hum. Genet.*, *46*(3), 428–433.

Kirby, D. M., McFarland, R., Ohtake, A., Dunning, C., Ryan, M. T., Wilson, C., et al. (2004). Mutations of the mitochondrial ND1 gene as a cause of MELAS. *J. Med. Gen.*, 41(10), 784–789. doi:10.1136/jmg.2004.020537

Moraes, C. T., Ciacci, F., Bonilla, E., Jansen, C., Hirano, M., Rao, N., et al. (1993). Two novel pathogenic mitochondrial DNA mutations affecting organelle number and protein synthesis. Is the tRNA(Leu(UUR)) gene an etiologic hot spot?. *J. Clin. Investig.*, 92(6), 2906–2915. doi:10.1172/JCI116913

Prezant, T. R., Agapian, J. V., Bohlman, M. C., Bu, X., Oztas, S., Qiu, W. Q., et al. (1993). Mitochondrial ribosomal RNA mutation associated with both antibiotic-induced and non-syndromic deafness. *Nat. Gen.*, 4(3), 289–294. doi:10.1038/ng0793-289

Santorelli, F. M., Tanji, K., Kulikova, R., Shanske, S., Vilarinho, L., Hays, A. P., and DiMauro, S. (1997). Identification of a novel mutation in the mtDNA ND5 gene associated with MELAS. *Biochem. Biophys. Res. Commun.*, 238(2), 326–328. doi:10.1006/bbrc.1997.7167

Sarzi, E., Brown, M. D., Lebon, S., Chretien, D., Munnich, A., Rotig, A., and Procaccio, V. (2007). A novel recurrent mitochondrial DNA mutation in ND3 gene is associated with isolated complex I deficiency causing Leigh syndrome and dystonia. *Am. J. Hum. Genet. A*, *143A*(1), 33–41. doi:10.1002/ajmg.a.31565

Seedorff T. (1985). The inheritance of Leber's disease. A genealogical follow-up study. *Acta Ophthalmol.*, 63(2), 135–145. doi:10.1111/j.1755-3768.1985.tb01526.x

Sweeney, M. G., Bundey, S., Brockington, M., Poulton, K. R., Winer, J. B., and Harding, A. E. (1993). Mitochondrial myopathy associated with sudden death in young adults and a novel mutation in the mitochondrial DNA leucine transfer RNA(UUR) gene. *Quarterly J. Med*, *86*(11), 709–713. doi:10.1093/oxfordjournals.qjmed.a068750

Tiranti, V., Chariot, P., Carella, F., Toscano, A., Soliveri, P., Girlanda, P., et al. (1995). Maternally inherited hearing loss, ataxia and myoclonus associated with a novel point mutation in mitochondrial tRNASer(UCN) gene. *Hum. Mol. Genet.*, 4(8), 1421–1427. doi:10.1093/hmg/4.8.1421

Wallace, D. C., Zheng, X. X., Lott, M. T., Shoffner, J. M., Hodge, J. A., Kelley, R. I., et al. (1988). Familial mitochondrial encephalomyopathy (MERRF): genetic, pathophysiological, and biochemical characterization of a mitochondrial DNA disease. *Cell*, *55*(4), 601–610. doi:10.1016/0092-8674(88)90218-8

Yoneda, M., Tanno, Y., Horai, S., Ozawa, T., Miyatake, T., and Tsuji, S. (1990). A common mitochondrial DNA mutation in the t-RNA(Lys) of patients with myoclonus epilepsy associated with ragged-red fibers. *Biochem. Int.*, 21(5), 789–796.

**Supplementary Table S5 References**

Alston, C. L., Morak, M., Reid, C., Hargreaves, I. P., Pope, S. A., Land, J. M., et al. (2010). A novel mitochondrial MTND5 frameshift mutation causing isolated complex I deficiency, renal failure and myopathy. *Neuromuscul. Disord.,* 20(2), 131–135. doi:10.1016/j.nmd.2009.10.010

Khusnutdinova, E., Gilyazova, I., Ruiz-Pesini, E., Derbeneva, O., Khusainova, R., Khidiyatova, I., et al. (2008). A mitochondrial etiology of neurodegenerative diseases: evidence from Parkinson's disease. *Ann. N. Y. Acad. Sci.*, 1147, 1–20. doi:10.1196/annals.1427.001

Nogueira, C., Silva, L., Pereira, C., Vieira, L., Leão Teles, E., Rodrigues, E., et al. (2019). Targeted next generation sequencing identifies novel pathogenic variants and provides molecular diagnoses in a cohort of pediatric and adult patients with unexplained mitochondrial dysfunction. *Mitochondrion*, 47:309-317. doi: 10.1016/j.mito.2019.02.006

Rucheton, B., Jardel, C., Filaut, S., Amador, M. D. M., Maisonobe, T., Serre, I., et al. (2020). Homoplasmic deleterious MT-ATP6/8 mutations in adult patients. *Mitochondrion*, 55, 64–77. doi:10.1016/j.mito.2020.08.004
