# Supplementary material for: The genetic landscape of mitochondrial diseases in the next-generation sequencing era: a Portuguese cohort study
Source: Front Cell Dev Biol. 2024 Feb 23;12:1331351. doi: 10.3389/fcell.2024.1331351 (PMC10920333; doi:10.3389/fcell.2024.1331351)
Supplement: Supplementary file 6 [file Table4.docx]

| Supplementary Table S4 – Pathogenic and likely pathogenic variants in mitochondrial DNA identified by next generation sequencing. | | | | | | | | | | | | | | | |
| --- | --- | --- | --- | --- | --- | --- | --- | --- | --- | --- | --- | --- | --- | --- | --- |
|  | | | | | | | | | | | | | | | |
| **Patient** | **Dx Age** | **Gender** | **Clinical presentation** | **Gene** | **Nucleotide change** | **Protein change** | **MTB** | **MC1** | **APG**  **2** | **MTIP** | **Conservation** | **Mitomap Frequency**  **(61168 FL)** | **Homolasmy/ Heterolasmy**  **Segregation** | **Status**  **(Mitomap [ClinGen])** | **References** |
| **P89** | 66y | M | Parkinsonism;  FHx of MD | *MT-RNR1* | m.1555A>G | - | - | - | - | - | 86.67% | 1.14e^-3^ | (B) Homo  - | Cfrm [P] | Prezant (1993) |
| **P90** | 62A | F | Deafness; cognitive delay; dystonic and cerebellar syndrome;  FHx of similar presentation. | *MT-RNR1* | m.1555A>G | - | - | - | - | - | 86.67% | 1.14e^-3^ | (B) Homo  - | Cfrm [P] | Prezant (1993) |
| **P91** | 55Y | F | Deafness; polyneuropathy. | *MT-RNR1* | m.1555A>G | - | - | - | - | - | 86.67% | 1.14e^-3^ | (B) Homo  - | Cfrm [P] | Prezant (1993) |
| **P92** | 2y | M | Severe cardiac dysfunction; hyperlactacidemia. | *MT-TL1* | m.3243A>G | - | - | - | - | CP | 97.78% | 1.7e^-4^ | (B) 10%  (M) 20%  *De novo* | Cfrm [P] | Goto (1990) |
| **P93** | 40y | F | Diabetes; nephropathy. | *MT-TL1* | m.3243A>G | - | - | - | - | CP | 97.78% | 1.7e^-4^ | (B) 30%  (BM) 36%  - | Cfrm [P] | Goto (1990) |
| **P94** | 54y | F | Mitochondrial disorder;  FHx of MD | *MT-TL1* | m.3243A>G | - | - | - | - | CP | 97.78% | 1.7e^-4^ | (B) 20%  - | Cfrm [P] | Goto (1990) |
| **P95** | 48y | M | Diabetes; deafness; nephropathy.  FHx of MIDD. | *MT-TL1* | m.3243A>G | - | - | - | - | CP | 97.78% | 1.7e^-4^ | (B) 20%  - | Cfrm [P] | Goto (1990) |
| **P96** | 42y ^†^ | M | MELAS phenotype. | *MT-TL1* | m.3243A>G | - | - | - | - | CP | 97.78% | 1.7e^-4^ | (B) 20%  - | Cfrm [P] | Goto (1990) |
| **P97** | 57y | M | Ischemic stroke; hyperlactacidemia; sensorineural deafness;  FHx of deafness. | *MT-TL1* | m.3243A>G | - | - | - | - | CP | 97.78% | 1.7e^-4^ | (B) 15%  - | Cfrm [P] | Goto (1990) |
| **P98** | 62y | M | Diabetes at young age; cardiac symptoms. | *MT-TL1* | m.3243A>G | - | - | - | - | CP | 97.78% | 1.7e^-4^ | (B) 10%  - | Cfrm [P] | Goto (1990) |
| **P99** | 61y | F | MIDD | *MT-TL1* | m.3243A>G | - | - | - | - | CP | 97.78% | 1.7e^-4^ | (B) 5%  - | Cfrm [P] | Goto (1990) |
| **P100** | 31y | F | Stroke. | *MT-TL1* | m.3243A>G | - | - | - | - | CP | 97.78% | 1.7e^-4^ | (B) 41%  - | Cfrm [P] | Goto (1990) |
| **P101** | 31y | F | Short stature; deafness; ataxia; epilepsy | *MT-TL1* | m.3243A>G | - | - | - | - | CP | 97.78% | 1.7e^-4^ | (B) 40%  - | Cfrm [P] | Goto (1990) |
| **P102** | 13A | M | Ataxia; epilepsy;  diabetes; stroke  FHx of deafness. | *MT-TL1* | m.3243A>G | - | - | - | - | CP | 97.78% | 1.7e^-4^ | (B) 55%  - | Cfrm [P] | Goto (1990) |
| **P103** | 12y | F | Myopathy. | *MT-TL1* | m.3251A>G | - | - | - | - | PB | 93.33% | - | (B) 70%  (M) 95% | Reported | Sweeney (1993) |
| **P104** | 21y | F | Myalgias; hyperlactacidemia. | *MT-TL1* | m.3271T>C | - | - | - | - | CP | 82.22% | - | (B) 40%  (M) 85%  Mother:  (B) 25% | Cfrm [P] | Goto (1991) |
| **P105** | 31y | M | MELAS phenotype. | *MT-TL1* | m.3271T>C | - | - | - | - | CP | 82.22% | - | (B) 25%  - | Cfrm [P] | Goto (1991) |
| **P106** | 10Y |  | Cardiomyopathy; diabetes; deafness; short stature; muscle weakness; hiperlactacidemia  FHx of MELAS | *MT-TL1* | m.3271T>C | - | - | - | - | CP | 82.22% | - | (B) 65%  Mother:  (B) 40% | Cfrm [P] | Goto (1991) |
| **P107** | 7y | F | Delayed psychomotor development; ataxia; hyperlactacidemia; MRI compatible with Leigh syndrome. | *MT-ND1* | m.3946G>A | p.Glu214Lys | D | D | **LP** | - | 100.00% | 2.0e^-5^ | (B) 70%  *De novo* | Reported | Kirby (2004) |
| **P108** | 27y | F | Myoclonus; ataxia; epileptic seizures. | *MT-TI* | m.4311G>A | - | - | - | - | LP | 93.33% | - | (B) 89%  (M)95%  Mother:  (B) 15% | - | This study |
| **P109** | 57y | F | Myoclonus epilepsy; ophthalmoplegia; ataxia; Ragged-Red-Fibers and COX deficiency. | *MT-TN* | m.5703G>A | - | - | - | - | CP | 8.89% | 2.0e^-5^ | (B) 16%  (M) 50%  - | Cfrm [P] | Moraes (1993) |
| **P110** | 9y | M | Leigh syndrome. | *MT-CO1* | m.6547T>C | p.Leu215Pro | D | D | **LP** | - | 100.00% | 7.0e^-5^ | (B) 50%  (M) 50% | Reported | Yoneda (1990) |
| **P111** | 69y | M | Chronic progressive external ophthalmoplegia; head drop syndrome; tetraparesia. | *MT-TS1* | m.7471insC | - | - | - | - | - | 4.44% | 1.2e^-4^ | (B) 40%  - | Cfrm [P] | Tiranti (1995) |
| **P112** | 70y | M | Gait abnormality; slimming; lipomas; dyslipidemia; maternal history of lipomas. | *MT-TK* | m.8344A>G | - | - | - | - | CP | 37.78% | 7.0e^-5^ | (B) 87%  - | Cfrm [P] | Wallace (1988) |
| **P113** | 19y | F | Delayed psychomotor development; sensorineural deafness; retinopathy; maculopathy; ataxia; polyneuropathy. | *MT-ATP6* | m.8993T>G | p.Leu156Arg | D | D | P | - | 97.78% | 1.0e^-4^ | (B) 95%  - | Cfrm [P] | Holt (1990) |
| **P114** | 44y | M | Retinitis pigmentosa;  FHx of Leigh syndrome. | *MT-ATP6* | m.8993T>G | p.Leu156Arg | D | D | P | - | 97.78% | 1.0e^-4^ | (B) 71%  - | Cfrm [P] | Holt (1990) |
| **P115** | 6m | M | Decreased citrulline in neonatal screening. | *MT-ATP6* | m.9176T>G | p.Leu217Arg | D | D | P | - | 100.00% | 2.0e^-5^ | (B) Homo  *De novo* | Cfrm [P] | Carrozzo (2000) |
| **P116** | 3y | M | Leigh syndrome. | *MT-ND3* | m.10197G>A | p.Ala47Thr | D | D | LP | - | 95.56% | 7.0e^-5^ | (M) Homo  - | Cfrm [P] | Sarzi (2007) |
| **P117** | 17y | M | Hereditary optic neuropathy. | *MT-ND4* | m.11778G>A | p.Arg340His | D | D | P | - | 100.00% | 3.15e^-3^ | (B) Homo  - | Cfrm [P] | Seedorff (1985) |
| **P118** | 74y | M | Bilateral optic atrophy. | *MT-ND4* | m.11778G>A | p.Arg340His | D | D | P | - | 100.00% | 3.15e^-3^ | (B) Homo  - | Cfrm [P] | Seedorff (1985) |
| **P119** | 28y | F | Myopathy; bilateral optic atrophy; pallidum calcifications; short stature and low weight. | *MT-TS2* | m.12213G>A | - | - | - | - | PP | 88.89% | - | (B) 25%  (M) 98%  *De novo* | . | This study |
| **P120** | 7y | M | Leigh syndrome. | *MT-ND5* | m.13513G>A | p.Asp393Asn | D | D | P | - | 100.00% | 2.0e^-5^ | (B) 58%  (M) 80%  *De novo* | Cfrm [P] | Santorelli (1997) |
| **P121** | 5y | F | Delayed psychomotor development; cardiomyopathy; hypotonia. | *MT-ND5* | m.13513G>A | p.Asp393Asn | D | D | P | - | 100.00% | 2.0e^-5^ | (B) 70%  - | Cfrm [P] | Santorelli (1997) |
| **P122** | 33y | F | MELAS phenotype. | *MT-ND5* | m.13513G>A | p.Asp393Asn | D | D | P | - | 100.00% | 2.0e^-5^ | (B) 25%  - | Cfrm [P] | Santorelli (1997) |
| **P123** | 2y | F | Failure to thrive; 2 episodes of ketoacidosis; organic acid profile alterations. | Δ^S^ | - | - | - | - | - | - | - | - | (B) Hetero  Size: 6,3Kb | [P] | This study |
| **P124** | 10y | F | Autism spectrum disorder;  hiperlactacidemia | Δ^S^ | - | - | - | - | - | - | - | - | (B) Hetero  Size: 10Kb | [P] | This study |
| **P125** | 1m | M | Hyperlactacidemia; hyperglycemia; hyperammonemia; pancytopenia; kidney dysfunction; ventricular hypertrophy. | Δ^S^ | - | - | - | - | - | - | - | - | (B) Hetero  Size: 5kb | [P] | This study |
| **P126** | 2y | M | Liver failure; hypoglycemia. | Δ^S^ | - | - | - | - | - | - | - | - | (B) Hetero  Size: 4kb | [P] | This study |

APG 2 (APOGEE: “B” benign, “LB” likely benign, “VUS” variant of unknown significance, “LP” likely pathogenic, “P” pathogenic); (B) – Blood; Cfrm [P] - Confirmed [Pathogenic]; (BM) - Buccal Mucosa; COX (Cytochrome c Oxidase); Dx (Diagnosis); F (Female); FHx (Family history); FL (Full length sequences); MELAS (Mitochondrial encephalomyopathy, lactic acidosis and stroke-like episodes); (M) – Muscle; m (months); M (Male); MC1 (MitoClass 1: “N” Neutral, “D” Damaging); MD – Mitochondrial Disorder; MIDD (Maternally Inherited Diabetes and Deafness); MRI (Magnetic resonance imaging); MTIP (MitoTIP: “LB” likely benign, “PB” possibly benign, “PP” possibly pathogenic, “LP” likely pathogenic, “CP” confirmed pathogenic); w (weeks); y (years); Δ^S^ (single large deletion of the mtDNA).

All references cited in this table can be consulted in DataSheet 2.
