# Supplementary material for: The genetic landscape of mitochondrial diseases in the next-generation sequencing era: a Portuguese cohort study
Source: Front Cell Dev Biol. 2024 Feb 23;12:1331351. doi: 10.3389/fcell.2024.1331351 (PMC10920333; doi:10.3389/fcell.2024.1331351)
Supplement: Supplementary file 7 [file DataSheet1.docx]

**Targeted nuclear genes panel**

*AARS2; ABCB7; ACAD9; ACO2; AGK; AIFM1; ALAS2; ALG13; APTX; ATP5A1; ATP5E; ATP5F1A; ATPAF2; AUH; BCS1L; BOLA3; C10ORF2; C12ORF65; C19ORF12; C9ORF116; CARS2; CHKB; CISD2; CLN3; CLN5; CLN6; CLN8; CLPB; COA3; COA5; COQ2; COQ4; COQ6; COQ8A; COQ9; COX10; COX14; COX15; COX20; COX4I2; COX5A; COX5B; COX6A1; COX6B1; COX7B; CTSD; CWF19L1; CYC1; DARS2; DGUOK; DLAT; DLD; DNA2; DNAJC19; DNAJC5; DNM1L; DOLK; EARS2; ECHS1; ELAC2; ETFA; ETFB; ETFDH; ETHE1; FARS2; FASTKD2; FBXL4; FDX1L; FLAD1; FOXRED1; GDAP1; GFER; GFM1; GLRX5; GTPBP3; HACE1; HARS2; HCCS; HIBCH; IARS2; IBA57; ISCU; KARS1; KCTD7; KIF5A; LARS; LARS2; LIAS; LIPT1; LRPPRC; LYRM4; LYRM7; MARS2; MEGF10; MFF; MFN1; MFN2; MFSD8; MGME1; MICU1; MPC1; MPI; MPV17; MRPL23; MRPL3; MRPL44; MRPL50; MRPL57; MRPS16; MRPS22; MTFMT; MTO1; MTPAP; NARS2; NDUFA1; NDUFA10; NDUFA11; NDUFA12; NDUFA2; NDUFA4; NDUFA9; NDUFAF1; NDUFAF2; NDUFAF3; NDUFAF4; NDUFAF5; NDUFAF6; NDUFB3; NDUFB9; NDUFS1; NDUFS2; NDUFS3; NDUFS4; NDUFS6; NDUFS7; NDUFS8; NDUFV1; NDUFV2; NFU1; NOTCH3; NUBPL; OPA1; OPA3; PANK2; PARS2; PC; PCK2; PDHA1; PDHB; PDHX; PDP1; PDSS1; PDSS2; PLD1; PMM2; PNPT1; POLG; POLG2; PPT1; PUS1; RARS2; RMND1; RNASEH1; RRM2B; SARS2; SCO1; SCO2; SDHA; SDHAF1; SDHAF2; SDHB; SDHC; SDHD; SERAC1; SLC19A3; SLC25A22; SLC25A3; SLC25A4; SLC25A42; SLC4A3; SLC52A1; SLC52A2; SLC52A3; SPATA5; SPG7; SPR; SUCLA2; SUCLG1; SURF1; TACO1; TARS2; TAFAZZIN; TIMM8A; TK2; TMEM126A; TMEM126B; TMEM70; TPK1; TPP1; TRMU; TRNT1; TSFM; TTC19; TUFM; TYMP; UQCRB; UQCRC2; UQCRQ; VARS2; WARS2; WDR45; WFS1; YARS2.*
